# In Silico Analysis of Fatty Acid Desaturases Structures in *Camelina sativa*, and Functional Evaluation of *Csafad7* and *Csafad8* on Seed Oil Formation and Seed Morphology

**DOI:** 10.3390/ijms221910857

**Published:** 2021-10-08

**Authors:** Nadia Raboanatahiry, Yongtai Yin, Kang Chen, Jianjie He, Longjiang Yu, Maoteng Li

**Affiliations:** Department of Biotechnology, College of Life Science and Technology, Huazhong University of Science and Technology, Wuhan 430074, China; nadia_raboana@hust.edu.cn (N.R.); yinyongtai@hust.edu.cn (Y.Y.); d201880535@hust.edu.cn (K.C.); hejianjie@hust.edu.cn (J.H.); yulongjiang@hust.edu.cn (L.Y.)

**Keywords:** *Camelina sativa*, fatty acid desaturase, structure, subcellular location, seed oil content, fatty acid profile

## Abstract

Fatty acid desaturases add a second bond into a single bond of carbon atoms in fatty acid chains, resulting in an unsaturated bond between the two carbons. They are classified into soluble and membrane-bound desaturases, according to their structure, subcellular location, and function. The orthologous genes in *Camelina sativa* were identified and analyzed, and a total of 62 desaturase genes were identified. It was revealed that they had the common fatty acid desaturase domain, which has evolved separately, and the proteins of the same family also originated from the same ancestry. A mix of conserved, gained, or lost intron structure was obvious. Besides, conserved histidine motifs were found in each family, and transmembrane domains were exclusively revealed in the membrane-bound desaturases. The expression profile analysis of *C. sativa* desaturases revealed an increase in young leaves, seeds, and flowers. *C. sativa* ω3-fatty acid desaturases *CsaFAD7* and *CsaDAF8* were cloned and the subcellular localization analysis showed their location in the chloroplast. They were transferred into *Arabidopsis thaliana* to obtain transgenic lines. It was revealed that the ω3-fatty acid desaturase could increase the C18:3 level at the expense of C18:2, but decreases in oil content and seed weight, and wrinkled phenotypes were observed in transgenic *CsaFAD7* lines, while no significant change was observed in transgenic *CsaFAD8* lines in comparison to the wild-type. These findings gave insights into the characteristics of desaturase genes, which could provide an excellent basis for further investigation for *C. sativa* improvement, and overexpression of ω3-fatty acid desaturases in seeds could be useful in genetic engineering strategies, which are aimed at modifying the fatty acid composition of seed oil.

## 1. Introduction

Fatty acid desaturases are enzymes, which turn a single bond of carbon atom into a double bond at specific positions of fatty acid hydrocarbon chains, resulting in an unsaturated bond between the two carbon atoms [1,2,3,4]. Desaturation occurs in the plastid and the endoplasmic reticulum (ER) with two different pathways [5,6]. Two classes of fatty acid desaturases (the soluble and the membrane-bound desaturase) have been identified [7]. Soluble desaturases (stearoyl-ACP [acyl-carrier-protein] desaturase SADs or FAB2) are located in the plastid and act on desaturation of stearoyl-ACP to oleoyl-ACP by adding a *Cis*-double bond between C9 and C10 of the carbon chain. Besides, membrane-bound desaturases are located in both plastid and ER, and they work on the desaturation of fatty acids, which are either turned into acyl-CoA via esterification or are bound to the glycerol part of glycerolipids [3]. Those membrane-bound desaturases are responsible for membrane lipid alteration and adjustment [8]. They include the ADS family (Acyl-lipid Δ9-desaturase) [9], the DES family (sphingolipids Δ4-desaturase) [10], and the FAD family. The FAD family includes the Δ12-desaturase/ω6-fatty acid desaturases FAD2 [11] and FAD6 [12], the Δ15-desaturase/ω3-fatty acid desaturases FAD3 [13], FAD7 [14] and FAD8 [15], and the Δ3-desaturase/palmitate desaturase FAD4 [16]. The SLD family (sphingolipids Δ8-desaturase) is also a membrane-bound desaturase, which acts on the desaturation of sphingolipids [17]. Otherwise, a common characteristic found in desaturases is the presence of conserved histidine motifs, which consist of a diiron binding site supporting the separation of the C-H bond to form water during the fatty acid desaturation [18,19]. Those genes encoding fatty acid desaturases are among the 600 genes encoding the proteins, which are implied in acyl-lipid formation in *A. thaliana* [20].

*Camelina sativa* is an allohexaploid *Brassicaceae* species (*n* = 20) [21,22,23], arising from the hybridization between *C. hispida* (diploid genome, *n* = 7) and *C. neglecta* (allotetraploid genome, *n* = 13) [24]. The model plant *Arabidopsis thaliana* also belongs to the *Brassicaceae* family, and it was estimated that the progenitors of *A. thaliana* and *C. sativa* separated ~17 MYA [23]. Both the *A. thaliana* and *C. sativa* genomes originated from the Ancestral Crucifer Karyotype (ACK), and both of them have experienced a genome rearrangement to develop their current genome structure [24,25,26,27]. *C. sativa* seeds contain about 36–47% oil, which is comparable to the oil content in rapeseed seeds, but of a higher quantity in comparison to that of soybean [28]. *C. sativa* provides biofuels for aviation, which reduces carbon dioxide emissions by 75% in comparison to petroleum [29]. With an abundance of long-chain hydrocarbons in its oil profile, *C. sativa* is qualified as a model crop for biofuels development [30].

Research findings have reported the improvement of oil content in *C. sativa* via genetic engineering. For instance, overexpression of *DGAT1* could increase the seed oil by 24% [31]; when being co-expressed with *GPD1*, the *DGAT1* oil increase was up to 13% [32], and co-expression with *MYB96A* resulted in an increase of 21% in fatty acid levels [33]. Other genes could also enhance the seed oil content in *C. sativa*, such as *AGG3* [34], *HMA3* (under heavy metal stress) [35], *WRI1* [36], the patatin-related phospholipase *AIIId* [37], *FAX1* and *ABCA9* [38] and *ZmLEC1* [39]. Otherwise, earlier research works on fatty acid desaturases were mostly focused on soluble desaturases [40] and *FAD2,* especially on their structure and expression [22,41] and function related to fatty acid alteration in *C. sativa* [42,43,44], and response to variations in temperature [45]. Previously, our team analyzed the function of *CsaFAD3* on seed phenotype and oil accumulation [46]. Particularly, in silico analysis of *C. sativa* desaturase genes, coupled with the study of expression and function associated with stress response, has been performed recently, using the NCBI gene bank for the identification of 24 desaturase genes [47]. However, a database of *Camelina* gene regulation, named CamRegBase, was established recently [48], which offers a broader view on *Camelina* genes, including their expression and regulation, and which is worth using to perform in silico analysis of desaturase genes in *C. sativa*, to compare them with the previous findings. Moreover, the gene and protein structures of the fatty acid desaturases in *C. sativa* have not been studied previously. Besides, the implications of the ω3-fatty acid desaturases *CsaFAD7* and *CsaFAD8* genes on seed oil and seed size have not clearly been understood until now, as *CsaFAD2* was often the focus of the previous research [22,41,42,43,44,45].

The purpose of this study was to identify and investigate the soluble and membrane-bound fatty acid desaturases in *C. sativa*, with the focus on their evolution and structure, and the implications of ω3-fatty acid desaturases on seed oil biosynthesis and seed size. Therefore, the following schemes were conducted: (1) the genes encoding fatty acid desaturases were identified in *C. sativa*, and the synteny and the evolutionary relationship of the fatty acid desaturases in *C. sativa* and *A. thaliana* were studied; (2) the structures of the fatty acid desaturase genes and proteins were examined; (3) the subcellular locations of the *C. sativa* fatty acid desaturases were uncovered; (4) the impact of modifying the genes encoding the ω3-fatty acid desaturases *CsaFAD7* and *CsaFAD8* on seed oil and seed morphology was reported. The current study would increase our knowledge on *C. sativa* fatty acid desaturases, which would serve as the basis for future research works on *C. sativa* breeding improvement.

## 2. Results

### 2.1. Synteny and Evolutionary Relationship of Fatty Acid Desaturase Genes in C. Sativa and A. thaliana

**Figure 1:**
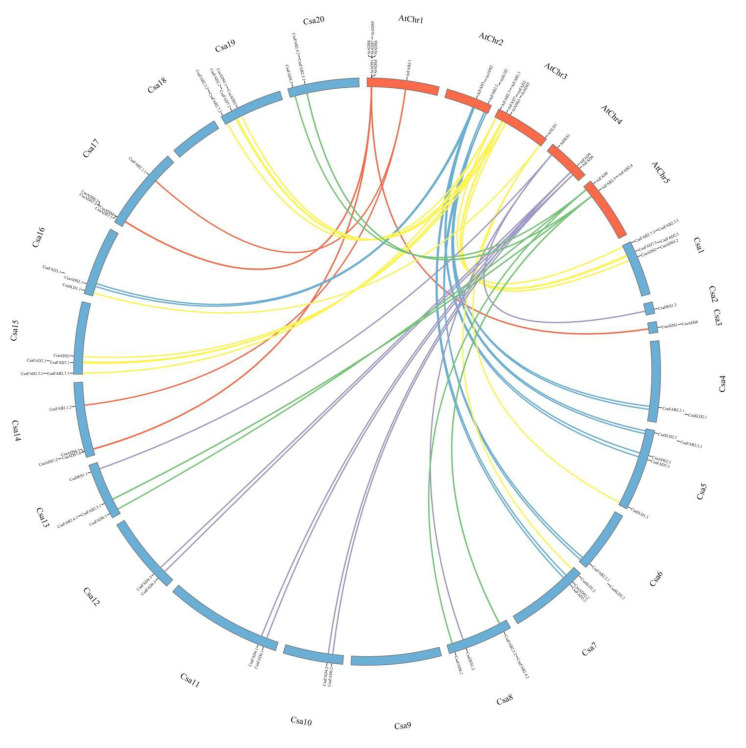
Synteny analysis of fatty acid desaturase genes in *A. thaliana* and *C. sativa.* The map was developed with TBtools software [49]. AtChr and Csa represent the chromosomes in *A. thaliana* and *C. sativa*, respectively. Gene names are arranged according to their position in the chromosomes, outside the circle.

In total, 62 fatty acid desaturase genes were identified, with 18 soluble *CsaFAB2* and 44 membrane-bound desaturase genes (17 *CsaADS*, 18 *CsaFAD*, 6 *CsaSLD* and 3 *CsaDES1*) (Table 1). The synteny of fatty acid desaturase genes in *A. thaliana* and *C. sativa* is illustrated in Figure 1. The copy number of fatty acid desaturase genes in *C. sativa* was higher in comparison to that of *A. thaliana*. Fatty acid desaturase genes were present in 18 among the 20 chromosomes of *C. sativa* (excluding chromosomes 9 and 18). The size of the proteins was larger in *C. sativa*, which might explain the difference in amino acid sequence identity.

**Figure 2:**
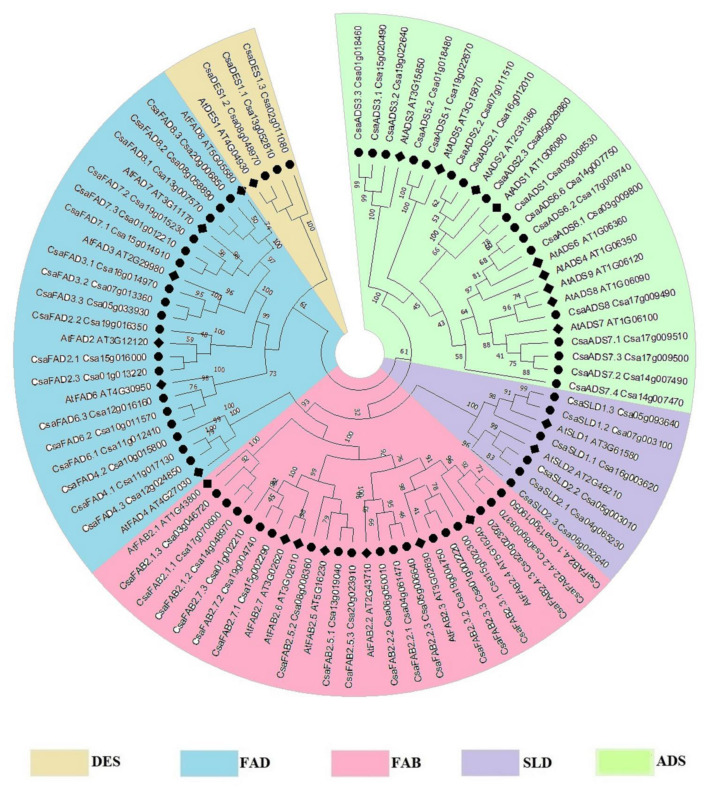
Evolutionary relationships of fatty acid desaturases. The tree was inferred using the Neighbor-Joining method [50]. The evolutionary distances were computed using the p-distance method [51] and are in the units of the number of amino acid differences per site. The analysis involved 87 amino acid sequences. Desaturase families were clustered into five classes, indicated by different colors. Numbers indicate the bootstrap value (%). Evolutionary analyses were conducted in MEGAX [52].

The evolutionary relationship of the fatty acid desaturases was made based on the amino acid sequences. Five clusters represented the five groups of fatty acid desaturases (Figure 2). Each group of fatty acid desaturase genes had moderately (bootstrap BS = 52%) to well (BS 100%) supported clades, which indicated the possible evolution of each group from a common origin. The divergence within each group occurred due to an alteration in amino acid sequences—some proteins were shorter or longer than the others—resulting in their evolution into separate clades. For example, ADS5 and ADS6 had 361 AA and 299 AA, respectively, which had led them to evolve into two distinct clades. It was also obvious that the longer proteins in *C. sativa* seemed to have evolved separately from the other members, which were closer to *A. thaliana* proteins rather than that other *C. sativa* proteins, as seen in CsaSLD1-Csa16g003620, which had 766 AA and was rather distant from the other CsaSLD1 (Csa07g003100 and Csa05g093640) in the phylogenetic tree. It was observed that some proteins were longer than other proteins of the same clade, for example, CsaADS7 (Csa14g007470) contained 898 AA, but its homolog in *A. thaliana* contained 299 AA, and other copies of CsaADS7 had 259-347 AA. There might be an annotation error with the gene ID Csa14g007470 (*CsaADS7*) on CamRegBase, which should be revised. This gene might be divided into three separate genes of about 300 AA, each. Nevertheless, it shared 80% similarity with AtADS7 and was found with the ADS7 clade in the phylogenetic tree.

### 2.2. Diversity in Gene and Protein Structure in C. sativa Fatty Acid Desaturases

**Figure 3:**
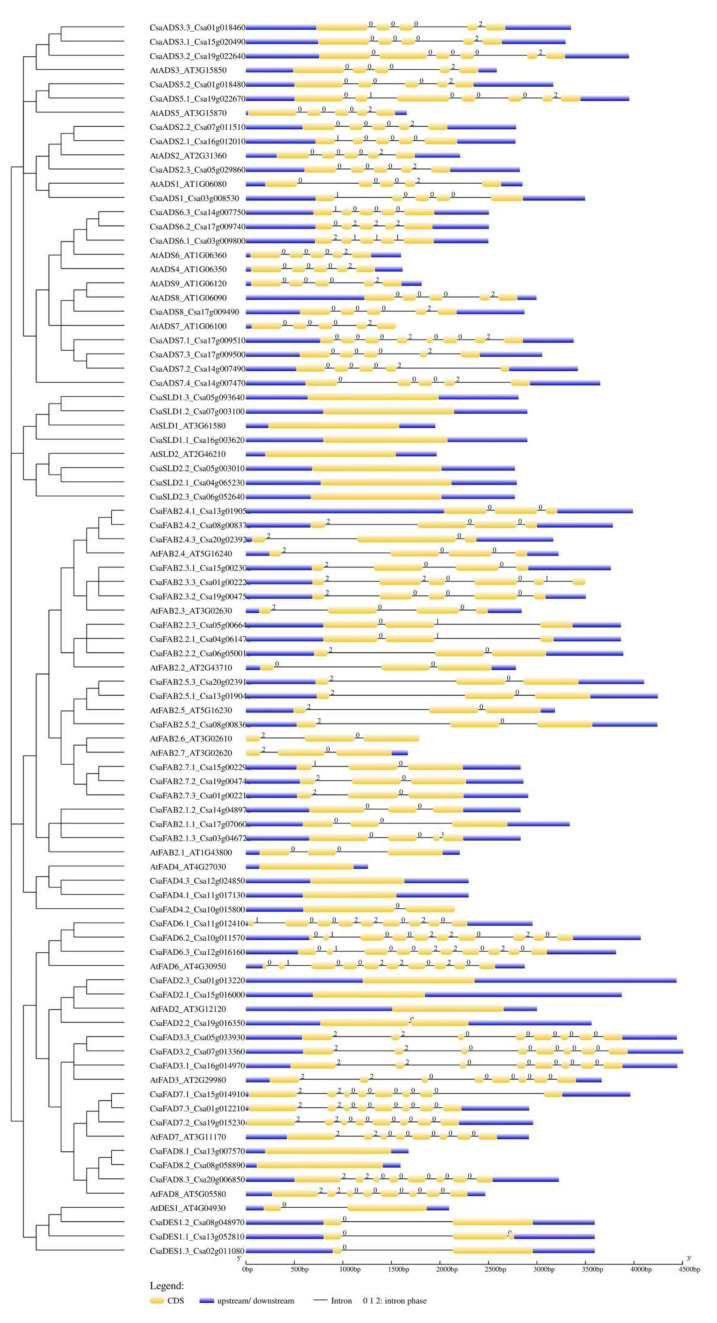
Gene structure of fatty acid desaturases in *A. thaliana* and *C. sativa.* Numbers indicate the intron phase.

Gene structural diversity could be characterized with the arrangement of introns–exons in individual fatty acid desaturase genes, and the type of introns (i.e., the phase in which they belonged). In phase 0 introns, no codon is disrupted; in phase 1 introns, a disruption between the first and second base of a codon occurs; and in phase 2 introns, a disruption between the second and third base of a codon occurs. It is very important to know which exon might be the target of alternative splicing since it might occur when introns of the same phase surround the exon, leading to what is called a symmetrical exon [53]. *C. sativa* desaturase genes experienced a mixture of gain and loss of intron–exon structure in comparison to those of *A. thaliana* (Figure 3). For example, *AtADS* contained five exons, but some *CsaADS* (e.g., *CsaADS3.2*-Csa19g022640) had gained one exon structure. Conversely, the *CsaFAD8.1*-Csa13g007570 and *CsaFAD8.2*-Csa08g058890 had lost all introns compared to their homolog *AtFAD8*. The *SLD* family seemed to be intronless. In all groups of fatty acid desaturase genes, most of the introns were of phase 0, and then phase 2, but phase 1 introns were few in number. Meanwhile, most of the genes contained exons, which were surrounded by two introns of the same type, such as in *CsaFAB2.1.1*-Csa17g070600, and might be a target for alternative splicing. The genes that had experienced a gain or loss of intron/exon were detected, possibly due to the pressure of evolution.

Protein domains are the essential structure that gives an identity to a protein and an indication of the protein’s function [54]. In the current study, 87 proteins of fatty acid desaturase from *A. thaliana* and *C. sativa* were subjected to a domain architecture analysis (Supplementary Table S1 and Figure S1). All of the analyzed proteins contained at least one fatty acid desaturase domain (FA_desaturase), except for the FAD4 class in *A. thaliana* and *C. sativa*, of which one or two B domains of TMEM were found. AtFAD4 (AT4G27030) was, though, recognized as a fatty acid (palmitate) desaturase in previous studies, despite lacking this FA_desaturase domain. The domain architecture of *C. sativa* desaturase differed with the number and family of the domain in each protein: FAB2 contained one lengthy FA_desaturase_2 domain (Ferritin-like, pfam03405), ADS had one to three FA_desaturase domains (pfam00487 and/or cl37993), and DES1 had one C-terminal FA_desaturase domain and one Lipid_DES domain in their *N*-terminal side. Similarly, the FAD family contained one C-terminal FA_desaturase domain (pfam00487), except for CsaFAD2.2 (Csa19g016350), which had two FA_desaturase domains, and an *N*-terminal DUF domain (cl13407 or cl15288), except for AtFAD6 (AT4G30950), CsaFAD6.2 (Csa10g011570), and CsaFAD6.3 (Csa12g016160), which lacked DUF domain. Finally, the SLD family contained a C-terminal FA_desaturase domain (pfam00487) and an *N*-terminal Cyt-b5 domain (pfam00173). CsaSLD1.1 (Csa16g003620) was an exception that it contained two FA_desaturase domains (pfam00487) and two Cyt-b5 domains (pfam00173). The FA_desaturase domains existed as three different types in the fatty acid desaturases (pfam03405, pfam00487, and cl37993). Pfam03405 is a Ferritin-like family and was exclusively found in the soluble fatty acid desaturase FAB2, whereas pfam00487 is a family, which belongs to the cl37993 superfamily, and they were solely found in the membrane-bound fatty acid desaturases.

Besides, two to ten and two to 14 transmembrane domains were found in membrane-bound desaturase by using SOSUI and TMHMM, respectively (Supplementary Figure S2). The FAD4 clade lacked any transmembrane domain in both *A. thaliana* and *C. sativa.* The numbers of transmembrane domains in each family varied from two to 14 in the ADS family, four to five in the DES family, one to nine in the FAD family, and in the SLD family, respectively. Besides, three histidine-enriched motifs were conserved in the soluble FAB2, while two to five histidine enriched motifs were found in membrane-bound desaturases (Table 2). It was remarkable to note the presence of motifs enriched with three consecutive histidines (HHHxH), exclusively in the ω3-fatty acid desaturases CsaFAD3, CsaFAD7, and CsaFAD8. Likewise, a motif that consists of HPMAGHFISEH was only found in CsaDES1. The divergence in gene structure, protein domain architecture, and histidine motifs were obvious and might explain the separate evolution of the fatty acid desaturases in *C. sativa*.

### 2.3. Expression Profile Analysis of C. sativa Desaturases Revealed an Increase in Expression in Young Leaves, Seeds, and Flowers

**Figure 4:**
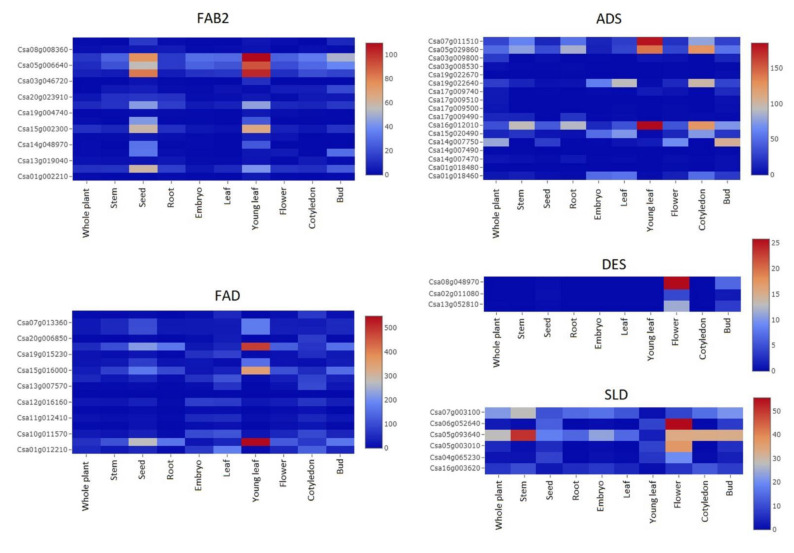
Expression pattern of fatty acid desaturase genes in C. sativa.

The expression profile of the five families of *C. sativa* desaturases was analyzed in a wild-type whole plant, stem, seed, root, embryo, leaf, young leaf, flower, cotyledon, and bud during the development phase (Figure 4). It was revealed that four *FAB2* genes (Csa06g050010, Csa05g006640, Csa04g061470, and Csa15g002300) were increased in young leaves and seeds, while *ADS2* genes’ expressions were increased in both young leaves and cotyledons, and *ADS6* (Csa14g007750) was increased in buds. *FAD2* genes were only increased in young leaves. One *DES1* (Csa08g048970) and two *SLD2* (Csa06g052640 and Csa05g003010) had increased expression in the flowers, while one *SLD1* (Csa05g093640) was increased in the stem, flower, and bud. This indicated that the accumulation of desaturase genes was associated with different tissues, and the expression differed among the desaturase genes families, which might add a further reason for their divergence in function, even within the same family with close a phylogenetic relationship.

### 2.4. C. sativa Fatty Acid Desaturases Were Detected in Three Intracellular Compartments

**Figure 5:**
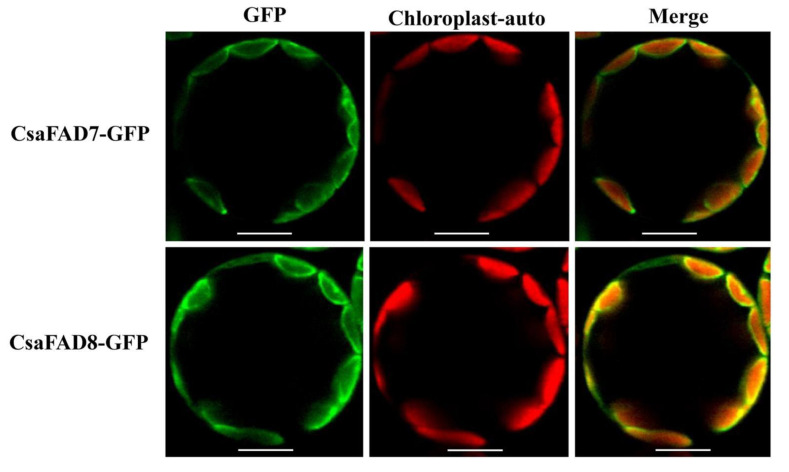
Subcellular location of CsaFAD7 and CsaFAD8. The *CsaFAD7* and *CsaFAD8* coding sequences were fused with EGFP and transiently transformed into *A. thaliana* mesophyll protoplast. GFP, the green fluorescent detection channel signal. Chloroplast-auto, the chlorophyll autofluorescence. Bar = 10 μm.

In silico analysis predicted that the soluble CsaFAB2 was located in chloroplast, which is similar to the membrane-bound CsaFAD4, CsaFAD6, CsaFAD7, and CsaFAD8. However, CsaFAD2 and CsaFAD3 were found in ER. In turn, CsaADS and CsaSLD were found in the plasma membrane (Supplementary Table S2). In our previous study, CsaFAD3 was located in the ER [46]. In the current study, the ω3-fatty acid desaturases CsaFAD7 and CsaFAD8 were transiently expressed in *A. thaliana* protoplast. As expected, the CsaFAD7 and CsaFAD8 were located in the chloroplast (Figure 5), which suggested that the ω3-fatty acid desaturases CsaFAD3, CsaFAD7, and CsaFAD8, which produced C18:3^CisΔ9,12,15^, existed in a functionally non-redundant form and played different roles in the evolutionary process.

### 2.5. Overexpression of CsaFAD7 Caused Seed Size and Oil Content Reduction in A. thaliana Seed but Not CsaFAD8

**Figure 6:**
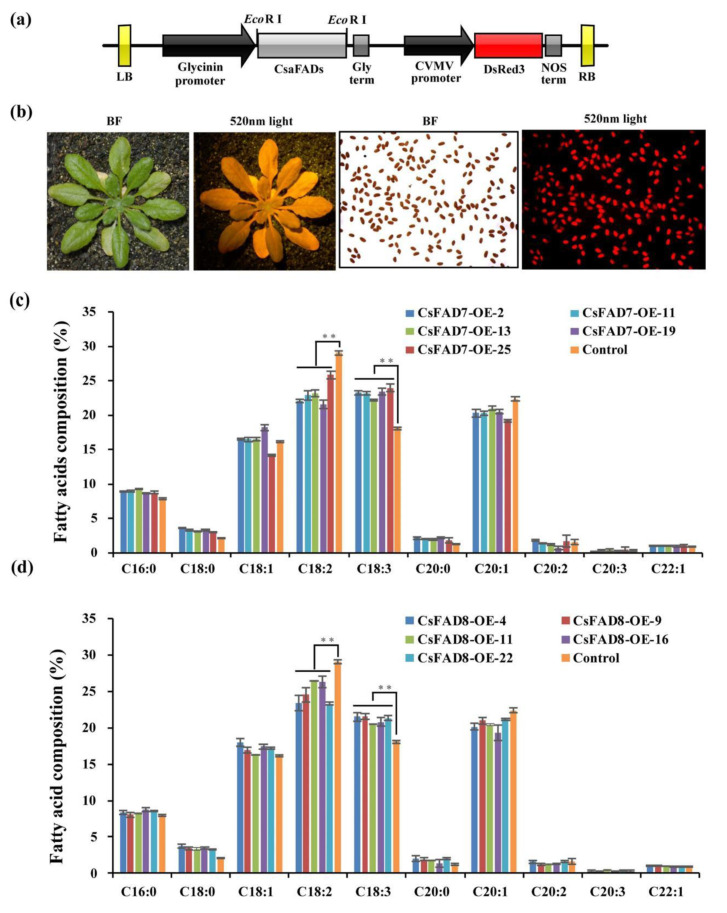
Transgenic screen of *CsaFAD7* and *CsaFAD8* seed-specific overexpression lines and fatty acid composition. (**a**) CsaFADs overexpression cassettes structure. LB, left border; RB, right border; Gly term, Glycinin terminator; NOS term, nopaline synthase terminator. (**b**) Screening of transgenic lines according to red fluorescence *DsRed3* marker. BF, bright field; 520 nm light, seedlings and seeds were activated by wavelength 520 nm light. (**c**,**d**) Fatty acid composition in *CsaFAD7* and *CsaFAD8* overexpression lines. T3 generation dry mature seeds were collected for GC analysis with 5 times repeat for each line. Asterisk represents a significant difference (*p* < 0.01, *n* = 5).

Previously, we revealed that the C18:3 production was negatively correlated with seed size and roundness, via overexpression of the *CsaFAD3* in *A. thaliana* seeds [46]. The same seed phenotypes were analyzed in the current study. Thus, the cDNA encoding the ω-3 fatty acid desaturases *CsaFAD7* and *CsaFAD8* were overexpressed in *A. thaliana Col-0.* The *CsaFAD7* and *CsaFAD8* coding sequences were driven by a seed-specific expression promoter of Glycinin (Figure 6a). Red fluorescent protein *DsRed3* was used as a selection marker, for screening the positive transgenic seed (Figure 6b). More than 20 T1 transgenic lines were screened according to the red fluorescent marker of each group. For further fatty acid analysis, five homozygous transgenic lines were selected from *CsaFAD7* and *CsaFAD8* overexpression lines, respectively. The expression levels of *CsaFAD7* and *CsaFAD8* genes in the transgenic lines were detected by q-PCR in the developing siliques, five days after flowering, and revealed a significantly higher level in the transgenic lines, i.e., three to seven times higher than those of the wild-type and the empty vector control group (Supplementary Figure S3). However, no significant difference was observed between the expression level of *CsaFAD7* and *CsaFAD8* in the transgenic lines.

A significant increase in C18:3^CisΔ9,12,15^ and a significant decrease in C18:2^Cis∆9,12^ in all lines transformed with *CsaFAD3* were reported previously [46]. In the current study, all of the transgenic lines transformed with *CsaFAD7* and *CsaFAD8* displayed a slight increase in C18:3 (~5.89% and ~3.25%, respectively, Supplementary Figure S4) and a decrease in C18:2 (~3.22% and ~5.80%, respectively) (Figure 6c,d). All these indicate that the ER located protein *CsaFAD3* contributed more in C18:3 production rather than the chloroplastic located proteins *CsaFAD7* and *CsaFAD8*. The C18:3 composition was higher in the *CsaFAD7* overexpression line than in *CsaFAD8*.

**Figure 7:**
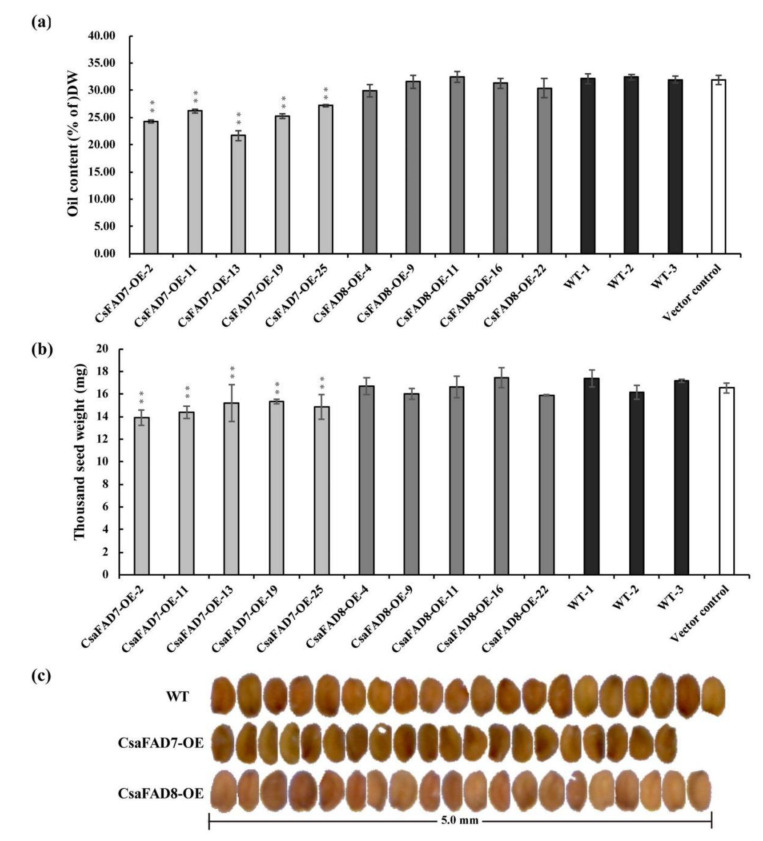
Oil content and seed morphology of *CsaFAD7* and *CsaFAD8* transgenic lines. (**a**) Oil content of *CsaFAD7* and *CsaFAD8* overexpression lines dry seeds. DW, dry weight. T3 generation dry mature seeds were collected for GC analysis with 5 repeats for each line. Asterisk represents a significant difference (*p* < 0.01, *n* = 5). (**b**) Thousand seed weight of *CsaFAD7* and *CsaFAD8* overexpression lines dry seeds. The dry mature seed size was measured with 5 repeats for each line. Asterisk represents a significant difference (*p* < 0.01, *n* = 5). Vector control, *pBinGlyRed3* transgenic lines under wild type background as control. (**c**) Seed appearance of *CsaFAD7*, *CsaFAD8* overexpression lines, and WT dry seeds.

Besides, the seed oil content significantly decreased in lines being transformed with *CsaFAD7* (~3.88%), while those transformed with *CsaFAD8* did not display any significant change in comparison with the wild-type (Figure 7a). Moreover, the thousand-seed weight (TSW) in *CsaFAD7* overexpression lines was also found lower than WT control and *CsaFAD8* overexpression lines. Those findings were similar to the *CsaFAD3* overexpression lines that have been reported previously [46], while the TSW of *CsaFAD8* overexpression lines did not show a significant decrease compared with the WT control (Figure 7b). The seed length to width value of the *CsaFAD7* transgenic lines showed a significant increase, while those of *CsaFAD8* were similar to the wild-type (Supplementary Figure S5). The seed of *CsaFAD7* transgenic lines displayed a wrinkled phenotype (Figure 7c), which was previously seldom found in *CsaFAD3* [46]. These results confirmed that overexpression of *CsaFAD7* caused abnormal oil accumulation while *CsaFAD8* did not. Moreover, the negative correlation between C18:3 accumulation and seed normalcy confirmed our previous studies. These findings suggest that *C. sativa* ω3-fatty acid desaturases play different roles in fatty acid accumulation and embryo development during the evolutionary process.

## 3. Discussion

### 3.1. Evolution of A. thaliana and C. sativa and their Respective Fatty Acid Desaturases

*C. sativa* fatty acid desaturase genes were identified based on homology with *A. thaliana* genes, and it was obvious that one gene in *A. thaliana* might have one to four copies of homologs in *C. sativa*, other genes had no homolog. A total of 62 fatty acid desaturase genes were found in CamRegBase during the current study, which was more numerous than the 24 genes found in NCBI [47]. In addition, some gene copies in *C. sativa* were longer than in *A. thaliana*—probably due to some errors, so this needs some revision—but they still had the FA_desaturase domain, which is common in fatty acid desaturases. The fact that *A. thaliana and C. sativa* both belong to the *Brassicaceae* family indicated that they both inherited the genomic blocks (GB) from the ACK, but the genome arrangement was different. *A. thaliana,* which has a genome size of 135Mb [55], has inherited a single copy of the 24 GB of the ACK [26]. However, *C. sativa* has been reported to have undergone a whole-genome triplication event [22], which indicated that *C. sativa* had a genome size and GB at least three times bigger in the size than those of *A. thaliana* [23]. The genome size of *C. sativa* was estimated to be ~750 to 785Mb [22,23,56], which was much larger than that of *A. thaliana*. Plant genome enlargement is the consequence of polyploidization and the expansion of transposable elements [57,58]. In general, during a polyploidization process, genome duplication/triplication, hybridization, and rearrangement occur, which increase the offspring genome size (gene copies) but also put pressure on chromosomes, which might lead to some gene losses. This phenomenon was reported in the polyploid genome of rapeseed (*Brassica napus*) [59,60,61,62], of which a mixture of retained, duplicated, lost, and converted genes (homologous exchange) or chromosomal segments were found in comparison to its progenitors [63]. Similarly, the genome of *C. sativa* experienced two polyploidy events from progenitors, of which repeated hybridization of closely related ancestry occurred, almost without any subgenome rearrangement in comparison to the parental genomes, conferring general stability [24]. A previous study reported that ~70% of the annotated genes in *Camelina* were syntenic to *A. thaliana* [23]. Particularly, it was estimated that genes involved in lipid metabolism were 217% higher in *C. sativa* in comparison to *A. thaliana*, and three copies of oil genes were generally retained in *C. sativa* [23], which was consistent with our findings, which showed that the majority of fatty acid desaturase genes were present as three copies in *C. sativa*. Seven duplicated genes were also found in fatty acid desaturase genes, which also contributed to the expansion of desaturase genes in *C. sativa*.

Then, a phylogenetic tree displayed five clusters corresponding to the five families of desaturase, indicating that they have evolved separately. The profile of the phylogenetic tree was comparable to those found in soybean [6], sunflower [64], cotton [65], and wheat [66]. Within the individual clade, orthologous genes of *A. thaliana* and *C. sativa* were clustered together. Focusing on the evolutionary relationship of each family of desaturase, diverse classes of the gene have evolved separately. In the soluble FAB2, the FAB2.1 clade seemed to separate earlier before the simultaneous emergence of the other members of the clade (FAB2.2-FAB2.7). Similarly, ADS3 appeared to diverge before the emergence of ADS5, and then ADS1 and ADS2. The clade of ADS4, ADS6 to ADS9 seemed to appear later. Additionally, the Δ12 fatty acid desaturases FAD6 and FAD2 seemed to separate earlier from the other clades and the Δ15 fatty acid desaturases FAD3, FAD7, and FAD8 emerged later. The Δ3 desaturase FAD4, which lacked FA_desaturase and transmembrane domains in our analysis, seemed to have evolved separately from the rest of the FAD family. Then, the two classes of the SLD family just diverged from each other and had evolved separately. In a broad view, some gene classes had more than one gene copy in those *C. sativa* desaturases, possibly due to duplication events during polyploidization, resulting in genes with different sequences [67]. Furthermore, the emergence of paralogous genes allows the growth of a subfunctionalization or a neofunctionalization [68,69]. Conversely, some genes were lost and this process is common during genome rearrangement, due to the pressure of evolution [70].

### 3.2. Conservation and Diversification in Structure of the Fatty Acid Desaturases

Structural analysis of fatty acid desaturases would help to unravel the previous evolutionary relationship between them. The conserved structure might assure the stability of the maintained function, while divergence might silence the original function or cause a new function to arise [71]. Gene structure analysis revealed a mixture of conservation, gain, and loss of intron in *C. sativa* fatty acid desaturases genes. The single intron gain or loss might be the outcome of a long evolution process [62]. Particularly, some genes, including *CsaSLD*, **CsaFAD4**, and some *CsaFAD2* and *CsaFAD8*, were intronless. Lack of intron was also observed in *SLD* and *FAD2* of rice [72] and grasses [73], and in *SLD*, *FAD2,* and *FAD4* of wheat [66], which is rather common in *SLD* genes [74]. In eukaryotes, intronless genes originate from horizontal gene transfer, from retrotransposon, or duplication of another intronless gene [75]. Besides, stress response genes such as *FAD2* [76] and *FAD8* [15,77] were reported to contain very few introns [78,79]. The presence of seven introns in *AtFAD8* and *CsaFAD8.3* was surprising in this analysis.

The protein domain of *C. sativa* desaturases mostly displayed a conserved structure, similar to what is found in *A. thaliana*. Only eight proteins displayed additional domains, which were different from those of *A. thaliana*. Protein domains are masterpieces that specify a protein structure and function [54,80,81], they are formed from an association of short polypeptides conglomerates [82] and evolve independently [83,84,85]. Protein domains were suggested to be more conserved during evolution than protein sequences [86,87]. Paralogous proteins often emerge from domain duplication [88,89], but also from divergence and recombination [54,90], as seen in the FAD family of which dissimilar domain architecture arose. Those domain recombinations lead to the emergence of more functions [89]. FA_desaturase was commonly seen in all desaturase genes under three different classes, which are specific to each family of desaturase. Even the same class of FA_desaturase showed divergence in the amino acid sequences in different desaturase families. Additional domains were found in some desaturases, such as DUF domains in the FAD family, which correspond to functionally uncharacterized proteins [91], except for FAD4, which was enriched with TMEM domains corresponding to a transmembrane protein in the Pfam database [92]. The SLD family also had an additional cytochrome-b5 domain on their structure. Cyt-B5 are electron transporters/donors for the desaturases [93,94]. Likewise, the DES family had a Lipid_DES domain, which is exclusively found in sphingolipid Δ4-desaturase proteins [95]. The lack of Lipid_DES in CsaDES1.3 was surprising as by homology, it was much closer to AtDES1 rather than other desaturase families; the sequence 1 to 24 AA in CsaDES1.3 was much different from AtDES1, CsaDES1.1, and CsaDES1.2, and the missing amino acids were also found leading to a shorter protein in CsaDES1.3, which was possibly the cause of the Lip_DES domain loss in this protein. A protein domain might change within its structure and function, and changes might also occur in the genome of different species [85,96]. Protein evolution is then the result of domain duplication, divergence, convergence, or fusion [89,97,98,99,100]. The earlier phylogenetic tree of desaturase genes was based on protein domains. The clear separation between the five families of desaturase was explained, the fact that the soluble and the membrane-bound desaturases are unrelated despite their similarities [18,101], and that they might originate from different ancestors [6]; those points were confirmed in our study.

A common characteristic in the soluble and the membrane-bound desaturases is the presence of histidine motif [18,102], and membrane-bound desaturases have a transmembrane domain in their structure [3,7]. Between two and 14 transmembrane domains were found in membrane-bound desaturases, except for FAD4, which lacked this structure in TMHMM and SOSUI analysis. Protein domain analysis revealed through the presence of TMEM domain was discovered, which indicates a transmembrane protein in Pfam database [92]. Transmembrane domains adopt an alpha-helix shape [103]. In eukaryotes, the length and amino acid sequence of the transmembrane domain was strongly linked to the intracellular compartmentation of the membrane proteins [104]. CsaADS, CsaDES1, and CsaSLD were located in the plasma membrane and they displayed a comparable amount of transmembrane domains unless for longer proteins, which had richer domains. Three histidine motifs were found in the soluble FAB2, and two of them, which were ENRHG and DEKRHE, were similar to the motifs found in peanut [6], rice [72], and wheat [66]. Likewise, the two histidine motifs which were found in the CsaDES family (HLEHH and HNEHH) were also found in peanut [6], rice [72], and wheat [66], whereas the motifs found in CsaFAD (HRTHH and HVIHH) were similar to those found in rice [72]. How well those motifs are conserved in different species indicated their important involvement in protein and enzyme functions [105].

### 3.3. C. sativa Desaturases Accumulation Was Associated with Different Cellular Compartmentations and Tissues

Fatty acid desaturases in *C. sativa* were accumulated in three subcellular compartments (chloroplast, plasma membrane, and ER), and in five different tissues (young leaves, flowers, buds, seeds, and cotyledons). Previously, Chi et al. analyzed the expression of desaturases in *A. thaliana* and soybean and revealed the expression of *AtSLD1* in flowers [6], similar to *CsaSLD1* in our findings. Similarly, *AtSLD1* was expressed in all organs, particularly in flowers, while *AtSLD2* was highly accumulated in flowers and siliques, but displayed a low expression level in leaves, stems, and roots [106]. By contrast, *AtFAB2* was reported to be highly expressed in flowers, root, stem, adult leaves, and cotyledons, while *CsaFAB2* was increased only in young leaves and seeds. *AtADS* was also reported to accumulate in seeds and flowers, while in our study *CsaADS* were expressed in young leaves, cotyledons, and buds. The other desaturase genes had low expression or no expression at all in *C. sativa*; however, Chi et al. found *AtFAD5*, *AtFAD6*, *AtFAD7* were highly expressed in cotyledons, stems, and leaves [6]. In a recent study, *AtFAD7* and *AtFAD8* were found to be expressed in leaves [107]. Besides, the difference in gene expression levels between *C. sativa* and *A. thaliana* might be due to differences in tissue age and environmental factors. A study on gene expression of desaturase in *C. sativa* revealed their variation according to stress conditions [47]. The accumulation of desaturase genes in *C. sativa* seemed to differ from those of *A. thaliana*, which indicated a possible divergence of biological function between them.

### 3.4. Seed-Specific Expression of the cDNA Encoding CsaFAD7 and CsaFAD8 Affected the Fatty Acid Composition of Seed Oil and the Seed Size in A. thaliana

FAD are key enzymes in the regulation of polyunsaturated fatty acid biosynthesis. Particularly, ω-3 fatty acid desaturases have been proven to use C18:2 as substrates to produce 18:3 in the plastid, where FAD7 and FAD8 are located, and in the ER where FAD3 are found [5]. In plants, the accumulation of 18:3 under the activity of ω-3 fatty acid desaturases, particularly *FAD3* has been demonstrated in several studies, such as in soybean [108,109], in rice [110], in olive [111,112,113], in peanut [114], in flax [115], in walnuts [116], in cowpea [117], in alfalfa [118], in peach aphid [119] and now in *C. sativa,* according to the current study.

Earlier, we could demonstrate that the overexpression of *CsaFAD3* caused seed abnormality and high accumulation of C18:3 [46], but this was not enough to understand the function of *C. sativa* ω-3 fatty acid desaturases on seed phenotypes. Thus, *CsaFAD7* and *CsaFAD8* functions were also analyzed to ensure our understanding. Moreover, *CsaFAD7* and *CsaFAD8* were more found to be located in different compartments than *CsaFAD3*, which increased our interest in uncovering the consequent phenotype. Although all three ω-3 fatty acid desaturases of *C. sativa* enhanced C18:3, *CsaFAD3* remarkably produced more C18:3 [46], in comparison to *CsaFAD7* and *CsaFAD8*. Differences between those three desaturases were found in their structure (amino acid sequences) of which *CsaFAD7* and *CsaFAD8* were very much similar in our analysis (~80% of identity), but also in their intracellular compartments, where *CsaFAD7* and *CsaFAD8* were located in the chloroplast, while *CsaFAD3* were located in the ER. This might explain the comparable performance of *CsaFAD7* and *CsaFAD8* on producing C18:3 accumulation. Moreover, desaturation of C18:2 to produce C18:3 occurs in the ER [5]. It might be possible that *CsaFAD3* promoted more accumulation of C18:3 due to their presence in the main location where C18:3 is produced, and *CsaFAD7* and *CsaFAD8* in the chloroplast might indirectly affect C18:3 production. Conversely, the seed of transgenic *CsaFAD3* [46] and *CsaFAD7* lines displayed a decrease in seed size, seed weight, and a wrinkled seed surface, while no obvious alteration was found in *CsaFAD8* transgenic lines. The underlying reason why the seed showed decreased oil content and seed size was still unclear. When the unusual fatty acid was produced in heterologous seeds, the seed oil content was also decreased according to the previous reports [120,121,122,123,124,125]. There were two hypotheses associated with this phenotype including the fatty acid β-oxidation [126] and the inhibition of fatty acid synthesis [121]. Those reports also suggested that different plant embryos have various concentrations of tolerance to unusual fatty acids. Recently, seed-specific overexpression of *Lesquerella FAD3* dramatically increased the C18:3 content and increased the seed size in soybean [127], while heterologous seed-specific overexpression of *CsaFAD7* in the *A. thaliana* embryo showed the reverse phenotype. Those reports and findings imply that the production and accumulation of C18:3 showed various tolerance in *A. thaliana* and soybean. Certainly, ω3-fatty acid desaturases play different roles in embryo development and oil accumulation, but the dissimilarity found between transgenic *CsaFAD7* and *CsaFAD8* on the seed surface was intriguing. Previously, the expression and activity of *FAD7* and *FAD8* in *A. thaliana* were compared to investigate their response to stress conditions [107]. *FAD7* had high promoter activity and was accumulated more in leaves, whereas *FAD8* displayed lower promoter activity, and was less expressed in leaves. Additionally, *FAD7* accumulated in response to wound and decreased with abscisic acid treatment, while *FAD8* concentrated in response to cold or jasmonate treatment, and attenuated at high temperature. In the current study, expressions and activities of *CsaFAD7* and *CsaFAD8* were also different; the reason why one caused abnormal phenotype might be found in the 20% identity difference between them, or due to some other factors. This needs further investigation.

In conclusion, the present study focused on soluble and membrane-bound fatty acid desaturases in *C. sativa*. Their evolution, structure, and subcellular location were studied, and their implication in oil biosynthesis was investigated. The close similarity between *A. thaliana* and *C. sativa* reflected their common origins, and the expansion of orthologous genes in *C. sativa* was the consequence of polyploidization. Despite acting as desaturases, the analyses of gene and protein domain structures confirmed that soluble and membrane-bound proteins were unrelated in our study, but paralogous genes within each family originated from a common ancestry. The histidine motifs, which are common in desaturases, were conserved in *C. sativa.* The subcellular location of fatty acid desaturases was similar to those of *A. thaliana*, which might suggest similar activity of proteins, but divergence might occur since protein function also depends on key amino acids sequences. As of now, it is clear that similarly to other ω-3 fatty acid desaturases of the other species, those of *C. sativa* could effectively accumulate alpha-linolenic acid. To the best of our knowledge, the current study was the first one to analyze both the soluble and membrane-bound desaturases in *C. sativa*, exploring the evolutionary, structural, and functional aspects. Our findings would be an excellent foundation for an in-depth understanding of the characteristics of fatty acid desaturases in *C. sativa*, and could be subjected to many aspects of investigations by using genetic engineering to discover new interesting functions for *C. sativa* trait improvement.

## 4. Materials and Methods

### 4.1. Plant Materials and Growth Conditions

The *Columbia* wild-type *A. thaliana (Col-0)* and transgenic lines were cultured at 16 h light/8 h dark (100 μm/m^2^/s) under constant temperature 23 °C. The position of control and transgenic lines used for lipid analysis were changed in the same tray every 5 days to make the plants grow in the same conditions.

### 4.2. Identification of Desaturase Genes in C. sativa

Identification of *FAD* genes in *C. sativa* was based on homology with the 25 genes identified in *A. thaliana* [6,20,65], using *Camelina* Gene Regulation Database or “CamRegBase” (http://camregbase.org/ accessed on 14 September 2021) [48]. The chromosomes’ location, the sequence, and the size of the genes were kindly provided by Professor Danny Schnell and Mr. Eric Maina of Michigan State University, USA. Gene synteny was drawn using TBtools software (https://github.com/CJ-Chen/TBtools accessed on 14 September 2021) [49].

### 4.3. Phylogenetic Analysis of Desaturase Genes

Phylogenetic analysis was made with protein sequences from *A. thaliana* and *C. sativa.* Alignment was performed with ClustalX software (http://www.clustal.org accessed on 14 September 2021) [128]. The phylogenic tree was unrooted and was inferred with Neighborhood Joining (NJ) [50]. The bootstrap consensus tree inferred from 1000 replicates was taken to represent the evolutionary history of the taxa being analyzed [129]. The evolutionary distances were computed using the Poisson Correction method [130] and were in the units of the number of amino acid substitutions per site. All ambiguous positions were removed for each sequence pair (pairwise deletion) [131,132]. Evolutionary analyses were conducted in MEGA X (https://www.megasoftware.net accessed on 14 September 2021) [52].

### 4.4. Gene Structure Analysis

The intron/exon structures of the desaturase genes in *C. sativa* were identified based on alignments of their coding sequences with their genomic sequences, and a diagram was obtained using the GSDS-Gene structure display server (https://gsds.cbi.pku.edu.cn/ accessed on 14 September 2021) [133].

### 4.5. Protein Domain Structure and Conserved Motif Analysis

Protein conserved domains in *C. sativa* desaturases were analyzed with the Batch CD-search tool in the NCBI database (http://www.ncbi.nlm.nih.gov/Structure/bwrpsb/bwrpsb.cgi accessed on 14 September 2021) [134], with CDSEARCH/oasis_pfam v3 as a source and an e-cut off value of 0.10. Analysis of transmembrane helix motif in membrane-bound desaturases was made using TMHMM 2.0 server based on a hidden Markov model (http://www.cbs.dtu.dk/services/TMHMM accessed on 14 September 2021) [135] and SOSUI (https://harrier.nagahama-i-bio.ac.jp/sosui/sosuiG/sosuigsubmit.html accessed on 14 September 2021) [136]. The conserved histidine motif of membrane-bound desaturases was observed via proteins alignment with Vector NTI Advanced 11.5.1 software. (Thermo Fisher Scientific, Waltham, MA, USA).

### 4.6. Expression Pattern of Desaturase Genes

The expression of each gene in the five families of desaturases was acquired from CamRegBase gene expression search tool (http://camregbase.org/data_search/gene accessed on 14 September 2021) [48], using gene ID as a query. The inquiry was made in normal tissues (whole plant, stem, seed, root, embryo, leaf, young leaf, flower, cotyledon, and bud) during development.

### 4.7. Subcellular Localization Analysis

The subcellular localization of fatty acid desaturase proteins was predicted using CELLO v.2.5 (subCELlular Localization predictor) server (http://cello.life.nctu.edu.tw/ accessed on 14 September 2021) [137]. Then, the CsaFADs coding sequence fusing with GFP tag was inserted into *pCambia1303* vector between *Spe I* and *BstE II* promoted by 35S promoter. The reconstructed vector and ER marker fused with cyan fluorescence protein was co-transformed into *A. thaliana* protoplasts as described previously [138,139]. The *A. thaliana* protoplasts were imaged by a confocal laser scanning microscopy FV1000 (Olympus, Japan). The images were analyzed with FV10-ASW software (Olympus, Japan).

### 4.8. Construction of CsaFAD7 and Csafad8 Seed-Specific Expression Vectors and Expression in A. thaliana

The coding sequences of *CsaFAD7* and *CsaFAD8* were inserted into the *pBinGlyRed3* vector in the *EcoRI* site, under the control of a seed-specific Glycinin promoter. The reconstructed vectors were transformed into *Agrobacterium tumefaciens GV3101* and transfected to *A. thaliana* with the dip floral method. T3 generation homozygous transgenic *A. thaliana* lines were collected for the lipid analysis. The developing siliques were collected five days after flowering in the transgenic lines and the wild-type for the q-PCR analysis, and Actin7 was used as an internal control.

### 4.9. Transgenic Seed Size and Lipid Analysis

For the seed size analysis, 5 to 10 mg T3 dry seeds were weighted and then spread out on a plate separately to make sure the individual grains could be distinguished without overlapping. The seed grain image was acquired by the scanner (Unis D6810, Beijing, China). The grain shape characters were calculated by the SC-G grain appearance quality image analysis system (WSeen, Hangzhou, China) developed by Hangzhou WSeen Detection Technology Co., Ltd., China [140,141,142].

Fatty acid composition and oil content analyses were performed according to the previous description [46]. Briefly, 5 mg of dried seeds were collected following 1.5 mL of 2.5% H_2_SO_4_ methanol solution (Sinopharm, Beijing, China) with 0.01% BHT (Sigma-Aldrich, Germany), 0.4 mL methylbenzene, and 200 µL of 2 mg/mL C17:0 (Sigma-Aldrich, Hamburg, Germany) as internal standard. The mixture was incubated at 90 °C for 1 h, 1.8 mL of deionized water, and 1 mL of hexane was added to the mixture. The supernatant was transferred for gas chromatography (GC) with flame ionization detection analysis (Agilent, CA, USA). The resolution of fatty acid methyl esters (FAMEs) in the supernatant was achieved with a DB-23 column (30 m length with 0.25 mm inner diameter and 0.25 µm thickness film; Agilent, CA, USA). The injection volume is 1 µL with a split ratio of 10:1. The carrier gas was with a flow rate of 1 mL/min with a pressure of 17.39 psi. The oven temperature was initiated from 150 °C for 3 min, then increased at 10 °C per minute to 240 ℃ and held for 8 min.

## Figures and Tables

**Table 1:** List of FAD genes in *A. thaliana* and *C. sativa*.

Group	*A. thaliana* (*n* = 5)				*C. sativa* (*n* = 20)			
	Gene Name	Gene ID	Chr	Size (AA)	Gene ID	Chr	Size (AA)	Identity (%)
**Soluble**	***FAB2.1***	AT1G43800	1	391	Csa17g070600	17	391	92
					Csa14g048970	14	391	92
					Csa03g046720	3	394	87
	***FAB2.2***	AT2G43710	2	401	Csa04g061470	4	401	98
					Csa06g050010	6	401	98
					Csa05g006640	5	466	97
	***FAB2.3***	AT3G02630	3	396	Csa15g002300	15	396	96
					Csa19g004750	19	396	97
					Csa01g002220	1	544	96
	***FAB2.4***	AT5G16240	5	394	Csa13g019050	13	333	94
					Csa08g008370	8	406	88
					Csa20g023920	20	427	89
	***FAB2.5***	AT5G16230	5	401	Csa13g019040	13	402	95
					Csa08g008360	8	425	95
					Csa20g023910	20	403	96
	***FAB2.6***	AT3G02610	3	411	*			
	***FAB2.7***	AT3G02620	3	403	Csa15g002290	15	412	90
					Csa19g004740	19	409	89
					Csa01g002210	1	411	89
**Membrane-bound**	***ADS1***	AT1G06080	1	305	Csa03g008530	3	923	97
	***ADS2***	AT2G31360	2	307	Csa16g012010	16	307	96
					Csa07g011510	7	307	96
					Csa05g029860	5	305	96
	***ADS3/FAD5***	AT3G15850	3	371	Csa15g020490	15	372	95
					Csa19g022640	19	537	95
					Csa01g018460	1	373	95
	***ADS4***	AT1G06350	1	300	*			
	***ADS5***	AT3G15870	3	361	Csa19g022670	19	584	80
					Csa01g018480	1	361	81
	***ADS6***	AT1G06360	1	299	Csa03g009800	3	299	92
					Csa17g009740	17	422	89
					Csa14g007750	14	299	92
	***ADS7***	AT1G06100	1	299	Csa17g009510	17	347	85
					Csa14g007490	14	259	81
					Csa17g009500	17	288	80
					Csa14g007470	14	898	80
	***ADS8***	AT1G06090	1	299	Csa17g009490	17	300	91
	***ADS9***	AT1G06120	1	299	*			
	***DES1***	AT4G04930	4	332	Csa13g052810	13	266	87
					Csa08g048970	8	337	94
					Csa02g011080	2	307	87
	***FAD2***	AT3G12120	3	383	Csa15g016000	15	385	96
					Csa19g016350	19	502	96
					Csa01g013220	1	384	97
	***FAD3***	AT2G29980	2	386	Csa16g014970	16	439	96
					Csa07g013360	7	387	97
					Csa05g033930	5	387	96
	***FAD4***	AT4G27030	4	323	Csa11g017130	11	321	90
					Csa10g015800	10	555	91
					Csa12g024850	12	323	91
	***FAD6***	AT4G30950	4	448	Csa11g012410	11	558	95
					Csa10g011570	10	445	95
					Csa12g016160	12	496	95
	***FAD7***	AT3G11170	3	446	Csa15g014910	15	448	90
					Csa19g015230	19	448	90
					Csa01g012210	1	448	90
	***FAD8***	AT5G05580	5	435	Csa13g007570	13	433	94
					Csa08g058890	8	433	94
					Csa20g006850	20	433	94
	***SLD1***	AT3G61580	3	449	Csa16g003620	16	766	95
					Csa07g003100	7	449	91
					Csa05g093640	5	450	93
	***SLD2***	AT2G46210	2	449	Csa04g065230	4	449	95
					Csa06g052640	6	449	96
					Csa05g003010	5	508	95

* The gene is absent or lost in the species.

**Table 2:** Histidine motifs of fatty acid desaturases in *A. thaliana* and *C. sativa*.

	Motifs	Location (AA)
**FAB2**	HxxxxH	133–136
	ENRHG	283–287
	DEKRHE	394–400
**ADS1**	HRNLAH	83–88
	HRYHH	120–124
	HNNHH	252–256
**ADS2**	HRNLAH	85–90
	HRYHH	122–126
	HNNHH	254–258
**ADS3**	HRYHH	354–358
	HNNHH	486–490
**ADS5**	HRNLSH	361–366
	HRNLSH	398–403
	HNNHH	530–534
**ADS6**	HRFHH	114–118
	HNNHH	369–373
**ADS7**	HRFHH	714–718
	HNNHH	951–955
**DES1**	HELSH	106-110
	HLEHH	143–147
	HPMAGHFISEH	238–248
**FAD2**	HECGHH	107–112
	HRRHH	143–147
	HVAHH	435–439
**FAD3**	HDCGH	154–158
	HHQNH	193–197
	HHHGH	314–318
	HVIHH	357–361
**FAD4**	HAWAH	465–469
	HAEHH	494–498
**FAD6**	HDCAH	319–323
	HDRHH	355–359
	HHTAPH	472–477
	HIPHH	515–519
**FAD7**	HDCGH	170–174
	HHQNH	209–213
	HHHGH	330–334
	HVIHH	373–377
**FAD8**	HDCGH	158–162
	HRTHH	198–198
	HHHGH	318–322
	HVIHH	361–365
**SLD**	HPGTAWHH	182–189
	HIKDFH	398–403
	HDSGH	477–481
	HNAHH	573–577
	HDPDLQH	585–591

## Data Availability

Not applicable.

## Supplementary Materials

The following are available online at https://www.mdpi.com/article/10.3390/ijms221910857/s1.

## References

[1] Harwood J.L., Stumpf P.K., Conn E.E. (1980). Plant acyl lipids: Structure, distribution, and analysis. Biochemistry of Plants.

[2] Stumpf P.K., Stumpf P.K., Conn E.E. (1980). Biosynthesis of saturated and unsaturated fatty acids. Biochemistry of Plants.

[3] Los D.A., Murata N. (1998). Structure and expression of fatty acid desaturases. Biochim. Biophys. Acta.

[4] Nakamura M.T., Nara T.Y. (2004). Structure, function, and dietary regulation of delta6, delta5, and delta9 desaturases. Annu. Rev. Nutr..

[5] Ohlrogge J., Browse J. (1995). Lipid biosynthesis. Plant Cell.

[6] Chi X.Y., Yang Q.L., Lu Y.D., Wang J.Y., Zhang Q.F., Pan L.J., Yu S. (2011). Genome-wide analysis of fatty acid desaturases in soybean (*Glycine max*). Plant Mol. Biol. Rep..

[7] Murphy D.J. (1999). Production of novel oils in plants. Curr. Opin. Biotechnol..

[8] Kargiotidou A., Deli D., Galanopoulou D., Tsaftaris A., Farmaki T. (2008). Low temperature and light regulate delta 12 fatty acid desaturases (FAD2) at a transcriptional level in cotton (*Gossypium hirsutum*). J. Exp. Bot..

[9] Smith M.A., Dauk M., Ramadan H., Yang H., Seamons L.E., Haslam R.P., Forseille L. (2013). Involvement of *Arabidopsis* ACYL-COENZYME A DESATURASE-LIKE2 (At2g31360) in the biosynthesis of the very-long-chain monounsaturated fatty acid components of membrane lipids. Plant Physiol..

[10] Kachroo A., Shanklin J., Whittle E., Lapchyk L., Hildebrand D., Kachroo P. (2007). The *Arabidopsis* stearoyl-acyl carrier protein-desaturase family and the contribution of leaf isoforms to oleic acid synthesis. Plant Mol. Biol..

[11] Okuley J., Lightner J., Feldmann K., Yadav N., Lark E. (1994). *Arabidopsis FAD2* gene encodes the enzyme that is essential for polyunsaturated lipid synthesis. Plant Cell.

[12] Falcone D.L., Gibson S., Lemieux B., Somerville C. (1994). Identification of a gene that complements an *Arabidopsis* mutant deficient in chloroplast ω6 desaturase activity. Plant Physiol..

[13] Arondel V., Lemieux I., Hwang S., Gibson H.M. (1992). Goodman and C.R. Somerville, Map-based cloning of a gene controlling omega-3 fatty acid desaturation in *Arabidopsis*. Science.

[14] Iba K., Gibson S., Nishiuchi T., Fuse T., Nishimura M., Arondel V., Somerville C. (1993). A gene encoding a chloroplast omega-3 fatty acid desaturase complements alterations in fatty acid desaturation and chloroplast copy number of the fad7 mutant of *Arabidopsis thaliana*. J. Biol. Chem..

[15] Gibson S., Arondel V., Iba K., Somerville C. (1994). Cloning of a temperature-regulated gene encoding a chloroplast ω-3 desaturase from *Arabidopsis thaliana*. Plant Physiol..

[16] Gao J., Ajjawi I., Manoli A., Sawin A., Xu C., Froehlich J.E., Benning C. (2009). Fatty acid desaturase4 of *Arabidopsis* encodes a protein distinct from characterized fatty acid desaturases. Plant J..

[17] Ryan P.R., Liu Q., Sperling P., Dong B., Franke S., Delhaize E. (2007). A higher plant Δ8 sphingolipid desaturase with a preference for (Z)-isomer formation confers aluminum tolerance to yeast and plants. Plant Physiol..

[18] Shanklin J., Cahoon E. (1998). Desaturation and related modifications of fatty acids. Ann. Rev. Plant Physiol. Plant Mol. Biol..

[19] López Alonso D., García-Maroto F., Rodríguez-Ruiz J., Garrido J., Vilches M. (2003). Evolution of the membrane-bound fatty acid desaturases. Biochem. Syst. Ecol..

[20] Li-Beisson Y., Shorrosh B., Beisson F., Andersson M.X., Arondel V., Bates P.D., Ohlrogge J. (2013). Acyl-lipid metabolism. Arabidopsis Book.

[21] Gehringer A., Friedt W., Luhs W., Snowdon R.J. (2006). Genetic mapping of agronomic traits in false flax (*Camelina sativa* subsp. *sativa*). Genome.

[22] Hutcheon C., Ditt R.F., Beilstein M., Comai L., Schroeder J., Goldstein E., Kiser J. (2010). Polyploid genome of *Camelina sativa* revealed by isolation of fatty acid synthesis genes. BMC Plant Biol..

[23] Kagale S., Koh C., Nixon J., Bollina V., Clarke W.E., Tuteja R., Parkin I.A. (2014). The emerging biofuel crop *Camelina sativa* retains a highly undifferentiated hexaploid genome structure. Nat. Commun..

[24] Mandáková T., Pouch M., Brock J.R., Al-Shehbaz I.A., Lysak M.A. (2019). Origin and evolution of diploid and allopolyploid *Camelina* genomes were accompanied by chromosome shattering. Plant Cell.

[25] Lysak M.A., Berr A., Pecinka A., Schmidt R., McBreen K., Schubert I. (2006). Mechanisms of chromosome number reduction in *Arabidopsis thaliana* and related *Brassicaceae* species. Proc. Natl. Acad. Sci. USA.

[26] Schranz M.E., Lysak M.A., Mitchell-Olds T. (2006). The ABC’s of comparative genomics in the *Brassicaceae*: Building blocks of crucifer genomes. Trends Plant Sci..

[27] Lysak M.A., Mandáková T., Schranz M.E. (2016). Comparative paleogenomics of crucifers: Ancestral genomic blocks revisited. Curr. Opin. Plant Biol..

[28] Moser B.R. (2012). Biodiesel from alternative oilseed feedstocks: *Camelina* and field pennycress. Biofuels.

[29] Shonnard D.R., Williams L., Kalnes T.M. (2010). *Camelina* derived jet fuel and diesel: Sustainable advanced biofuels. Environ. Prog. Sustain. Energy.

[30] Iskandarov U., Kim H.J., Cahoon E.B., McCann M.C., Buckeridge M.S., Carpita N.C. (2014). *Camelina*: An emerging oilseed platform for advanced biofuels and bio-based materials. Plants and Bioenergy.

[31] Kim H., Park J.H., Kim D.J., Kim A.Y., Su M.C. (2016). Functional analysis of diacylglycerol acyltransferase1 genes from *Camelina sativa* and effects of *CsDGAT1B* overexpression on seed mass and storage oil content in *C. sativa*. Plant Biotechnol. Rep..

[32] Chhikara S., Abdullah H.M., Akbari P., Schnell D., Dhankher O.P. (2017). Engineering *Camelina sativa* (L.) *Crantz* for enhanced oil and seed yields by combining diacylglycerol acyltransferase1 and glycerol-3-phosphate dehydrogenase expression. Plant Biotechnol. J..

[33] Kim R.J., Kim H.U., Suh M.C. (2019). Development of *Camelina* enhanced with drought stress resistance and seed oil production by co-overexpression of MYB96A and DGAT1C. Ind. Crops Prod..

[34] Choudhury S.R., Riesselman A.J., Sona Pandey S. (2014). Constitutive or seed-specific overexpression of *Arabidopsis* G-protein c subunit 3 (AGG3) results in increased seed and oil production and improved stress tolerance in *Camelina sativa*. Plant Biotechnol. J..

[35] Park W., Feng Y., Kim H., Suh M.C., Ahn S.J. (2015). Changes in fatty acid content and composition between wild type and CsHMA3 overexpressing *Camelina sativa* under heavy-metal stress. Plant Cell Rep..

[36] An D., Suh M.C. (2015). Overexpression of *Arabidopsis* WRI1 enhanced seed mass and storage oil content in *Camelina sativa*. Plant Biotechnol. Rep..

[37] Li M., Wei F., Tawfall A., Tang M., Saettele A., Wang X. (2015). Overexpression of patatin-related phospholipase AIIIδ altered plant growth and increased seed oil content in *Camelina*. Plant Biotechnol. J..

[38] Cai G., Wang G., Kim S.C., Li J., Zhou Y., Wang X. (2021). Increased expression of fatty acid and ABC transporters enhances seed oil production in *Camelina*. Biotechnol. Biofuels.

[39] Zhu Y., Xie L., Chen G.Q., Lee M.Y., Loque D., Scheller H.V. (2018). A transgene design for enhancing oil content in *Arabidopsis* and *Camelina* seeds. Biotechnol. Biofuels.

[40] Rodríguez M.F., Sánchez-García A., Salas J.J., Garcés R., Martínez-Force E. (2015). Characterization of soluble acyl-ACP desaturases from *Camelina sativa*, *Macadamia tetraphylla* and *Dolichandra unguis-cati*. J. Plant Physiol..

[41] Kang J., Snapp A.R., Lu C. (2011). Identification of three genes encoding microsomal oleate desaturases (FAD2) from the oilseed crop *Camelina sativa*. Plant Physiol. Biochem..

[42] Kim H., Go Y.S., Kim A.Y., Lee S., Kim K.N., Lee G.J., Suh M.C. (2014). Isolation and functional analysis of three microsomal delta-12 fatty acid desaturase genes from *Camelina sativa* (L.) cv. CAME. J. Plant Biotechnol..

[43] Jiang W.Z., Henry I.M., Lynagh P.G., Comai L., Cahoon E.B., Weeks D.P. (2017). Significant enhancement of fatty acid composition in seeds of the allohexaploid, *Camelina sativa*, using CRISPR/Cas9 gene editing. Plant Biotechnol. J..

[44] Morineau C., Bellec Y., Tellier F., Gissot L., Kelemen Z., Nogué F., Faure J.D. (2017). Selective gene dosage by CRISPR-Cas9 genome editing in hexaploid *Camelina sativa*. Plant Biotechnol. J..

[45] Rodríguez-Rodríguez M.F., Salas J.J., Venegas-Calerón M., Garcés R., Martínez-Force E. (2016). Molecular cloning and characterization of the genes encoding a microsomal oleate Δ12 desaturase (CsFAD2) and linoleate Δ15 desaturase (CsFAD3) from *Camelina sativa*. Ind. Crops Prod..

[46] Yin Y., Guo Z., Chen K., Tian T., Tan J., Chen X., Li M. (2020). Ultra-high α-linolenic acid accumulating developmental defective embryo was rescued by lysophosphatidic acid acyltransferase 2. Plant J..

[47] Ahmadizadeh M., Rezaee S., Heidari P. (2020). Genome-wide characterization and expression analysis of fatty acid desaturase gene family in *Camelina sativa*. Gene Rep..

[48] Gomez-Cano F., Carey L., Lucas K., García Navarrete T., Mukundi E., Lundback S., Grotewold E. (2020). CamRegBase: A gene regulation database for the biofuel crop, *Camelina sativa*. Database.

[49] Chen C., Chen H., Zhang Y., Thomas H.R., Frank M.H., He Y., Xia R. (2020). TBtools: An integrative toolkit developed for interactive analyses of big biological data. Mol. Plant.

[50] Saitou N., Nei M. (1987). The neighbor-joining method: A new method for reconstructing phylogenetic trees. Mol. Biol. Evol..

[51] Nei M., Kumar S. (2000). Molecular Evolution and Phylogenics.

[52] Kumar S., Stecher G., Li M., Knyaz C., Tamura K. (2018). MEGA X: Molecular evolutionary genetics analysis across computing platforms. Mol. Biol. Evol..

[53] Choudhuri S. (2014). Chapter 1-Fundamentals of Genes and Genomes. Bioinformatics for Beginners.

[54] Itoh M., Nacher J.C., Kuma K., Goto S., Kanehisa M. (2007). Evolutionary history and functional implications of protein domains and their combinations in eukaryotes. Genome Biol..

[55] The Arabidopsis Genome Initiative (2000). Analysis of the genome sequence of the flowering plant *Arabidopsis thaliana*. Nature.

[56] Berti M., Gesch R., Eynck C., Anderson J., Cermak S. (2016). *Camelina* uses, genetics, genomics, production, and management. Ind. Crops Prod..

[57] Kumar A., Bennetzen J.L. (1999). Plant retrotransposons. Annu. Rev. Genet..

[58] Bennetzen J.L. (2002). Mechanisms and rates of genome expansion and contraction in flowering plants. Genetica.

[59] Lagercrantz U. (1998). Comparative mapping between *Arabidopsis thaliana* and *Brassica nigra* indicates that *Brassica* genomes have evolved through extensive genome replication accompanied by chromosome fusions and frequent rearrangements. Genetics.

[60] Lysak M.A., Koch M.A., Pecinka A., Schubert I. (2005). Chromosome triplication found across the tribe *Brassiceae*. Genome Res..

[61] Parkin I.A., Gulden S.M., Sharpe A.G., Lukens L., Trick M., Osborn T.C., Lydiate D.J. (2005). Segmental structure of the *Brassica napus* genome based on comparative analysis with *Arabidopsis thaliana*. Genetics.

[62] Cheng F., Wu J., Wang X. (2014). Genome triplication drove the diversification of Brassica plants. Hortic. Res..

[63] Chalhoub B., Denoeud F., Liu S., Parkin I.A., Tang H., Wang X., Chiquet J., Belcram H., Tong C., Samans B. (2014). Early allopolyploid evolution in the post-Neolithic *Brassica napus* oilseed genome. Science.

[64] Celik Altunoglu Y., Unel N.M., Baloglu M.C., Ulu F., Can T.H., Cetinkaya R. (2018). Comparative identification and evolutionary relationship of fatty acid desaturase (FAD) genes in some oil crops: The sunflower model for evaluation of gene expression pattern under drought stress. Biotechnol. Biotechnol. Equip..

[65] Liu W., Li W., He Q., Daud M.K., Chen J., Zhu S. (2015). Characterization of 19 genes encoding membrane-bound fatty acid desaturases and their expression profiles in *Gossypium raimondii* under low temperature. PLoS ONE.

[66] Hajiahmadi Z., Abedi A., Wei H., Sun W., Ruan H., Zhuge Q., Movahedi A. (2020). Identification, evolution, expression, and docking studies of fatty acid desaturase genes in wheat (*Triticum aestivum* L.). BMC genomics.

[67] Zhang J.Z. (2003). Evolution by gene duplication: An update. Trends Ecol. Evol..

[68] Rastogi S., Liberles D.A. (2005). Subfunctionalization of duplicated genes as a transition state to neofunctionalization. BMC Evol. Biol..

[69] Freeling M., Scanlon M.J., Fowler J.E. (2015). Fractionation and subfunctionalization following genome duplications: Mechanisms that drive gene content and their consequences. Curr. Opin. Genet. Dev..

[70] Hu T.T., Pattyn P., Bakker E.G., Cao J., Cheng J.F., Clark R.M., Guo Y.L. (2011). The *Arabidopsis lyrata* genome sequence and the basis of rapid genome size change. Nat. Genet..

[71] Cao J., Shi F. (2012). Evolution of the RALF gene family in plants: Gene duplication and selection patterns. Evol. Bioinf..

[72] Chen C., Yang J., Tong H., Li T., Wang L., Chen H. (2019). Genome-wide analysis of fatty acid desaturase genes in rice (*Oryza sativa* L.). Sci. Rep..

[73] Díaz M.L., Cuppari S., Soresi D., Carrera A. (2018). In silico analysis of fatty acid desaturase genes and proteins in grasses. Appl. Biochem. Biotechnol..

[74] Sperling P., Zahringer U., Heinz E. (1998). A sphingolipid desaturase from higher plants. Identification of a new cytochrome b5 fusion protein. J. Biol. Chem..

[75] Huang W., Xian Z., Kang X., Tang N., Li Z. (2015). Genome-wide identification, phylogeny and expression analysis of GRAS gene family in tomato. BMC Plant Biol..

[76] Zhang J., Liu H., Sun J., Li B., Zhu Q., Chen S., Zhang H. (2012). *Arabidopsis* fatty acid desaturase fad2 is required for salt tolerance during seed germination and early seedling growth. PLoS ONE.

[77] Zhang M., Barg R., Yin M., Gueta-Dahan Y., Leikin-Frenkel A., Salts Y., Ben-Hayyim G. (2005). Modulated fatty acid desaturation via overexpression of two distinct ω-3 desaturases differentially alters tolerance to various abiotic stresses in transgenic tobacco cells and plants. Plant J..

[78] Lan T., Gao J., Zeng Q.Y. (2013). Genome-wide analysis of the LEA (late embryogenesis abundant) protein gene family in Populus trichocarpa. Tree Genet. Genom..

[79] Xie D.W., Wang X.N., Fu L.S., Sun J., Zheng W., Li Z.F. (2015). Identification of the trehalose-6-phosphate synthase gene family in winter wheat and expression analysis under conditions of freezing stress. J Genet..

[80] Janin J., Wodak S.J. (1983). Structural domains in proteins and their role in the dynamics of protein function. Prog. Biophys. Mol. Biol..

[81] Han J.H., Batey S., Nickson A.A., Teichmann S.A., Clarke J. (2007). The folding and evolution of multidomain proteins. Nat. Rev. Mol. Cell Biol..

[82] Lupas A.N., Ponting C.P., Russell R.B. (2001). On the evolution of protein folds: Are similar motifs in different protein folds the result of convergence, insertion, or relics of an ancient peptide world?. J. Struct. Biol..

[83] Ponting C.P., Russell R.R. (2002). The natural history of protein domains. Annu. Rev. Biophys. Biomol. Struct..

[84] Vogel C., Bashton M., Kerrison N.D., Chothia C., Teichmann S.A. (2004). Structure, function and evolution of multidomain proteins. Curr. Opin. Struct. Biol..

[85] Yang S., Bourne P.E. (2009). The evolutionary history of protein domains viewed by species phylogeny. PLoS ONE.

[86] Wang M., Caetano-Anollés G. (2009). The evolutionary mechanics of domain organization in proteomes and the rise of modularity in the protein world. Structure.

[87] Illergård K., Ardell D.H., Elofsson A. (2009). Structure is three to ten times more conserved than sequence—a study of structural response in protein cores. Proteins Struct. Funct. Bioinforma..

[88] Lynch M., Conery J.S. (2000). The evolutionary fate and consequences of duplicate genes. Science.

[89] Vogel C., Teichmann S.A., Pereira-Leal J. (2005). The relationship between domain duplication and recombination. J. Mol. Biol..

[90] Grishin N.V. (2001). Fold change in evolution of protein structures. J. Struct. Biol..

[91] Bateman A., Coggill P., Finn R.D. (2010). DUFs: Families in search of function. Acta Crystallogr. Sect. F Struct. Biol. Cryst. Commun..

[92] Lodish H., Berk A., Zipursky S., Freeman W.H. (2000). Insertion of Membrane Proteins into the ER Membrane.

[93] Dailey H.A., Strittmatter P. (1980). Characterization of the interaction of amphipathic cytochrome b5 with stearoyl coenzyme A desaturase and NADPH:cytochrome P-450 reductase. J. Biol. Chem..

[94] Mitchell A.G., Martin C.E. (1995). A novel cytochrome b5-like domain is linked to the carboxyl terminus of the *Saccharomyces cerevisiae* v-9 fatty acid desaturase. J. Biol. Chem..

[95] Ternes P., Franke S., Zähringer U., Sperling P., Heinz E. (2002). Identification and characterization of a sphingolipid delta 4-desaturase family. J. Biol. Chem..

[96] Scheeff E.D., Bourne P.E. (2005). Structural evolution of the protein kinase-like superfamily. PLoS Comput. Biol..

[97] Gough J. (2005). Convergent evolution of domain architectures (is rare). Bioinformatics.

[98] Kummerfeld S.K., Teichmann S.A. (2005). Relative rates of gene fusion and fission in multi-domain proteins. Trends Genet..

[99] Björklund A.K., Ekman D., Elofsson A. (2006). Expansion of protein domain repeats. PLoS Comput. Biol..

[100] Vogel C., Morea V. (2006). Duplication, divergence and formation of novel protein topologies. Bioessays.

[101] Somerville C., Browse J. (1996). Dissecting desaturation: Plants prove advantageous. Trends Cell Biol..

[102] Xue Y., Chen B., Win A.N., Fu C., Lian J., Liu X., Wang R., Zhang X., Chai Y. (2018). Omega-3 fatty acid desaturase gene family from two ω-3 sources, *Salvia hispanica* and *Perilla frutescens*: Cloning, characterization and expression. PLoS ONE.

[103] Engelman D.M., Steitz T.A., Goldman A. (1986). Identifying nonpolar transbilayer helices in amino acid sequences of membrane proteins. Annu. Rev. Biophys. Biophys. Chem..

[104] Sharpe H.J., Stevens T.J., Munro S. (2010). A comprehensive comparison of transmembrane domains reveals organelle-specific properties. Cell.

[105] Hashimoto K., Yoshizawa A.C., Okuda S., Kuma K., Goto S., Kanehisa M. (2008). The repertoire of desaturases and elongases reveals fatty acid variations in 56 eukaryotic genomes. J. Lipid Res..

[106] Chen M., Markham J.E., Cahoon E.B. (2012). Sphingolipid Δ8 unsaturation is important for glucosylceramide biosynthesis and low-temperature performance in *Arabidopsis*. Plant J..

[107] Soria-Garcïa Ï.N., Rubio M.A.C., Lagunas B., Lï Pez-Gomollï N.S., Lujï N.M.A.L.Ï.N., Dï Az-Guerra R.L. (2019). Tissue Distribution and Specific Contribution of *Arabidopsis* FAD7 and FAD8 plastid desaturases to the JA- and ABA-mediated cold stress or defense responses. Plant Cell Physiol..

[108] Damude H.G., Zhang H., Farrall L., Ripp K.G., Tomb J.F., Hollerbach D., Yadav N.S. (2006). Identification of bifunctional Δ12/ω-3 fatty acid desaturases for improving the ratio of ω-3 to ω-6 fatty acid in microbes and plants. Proc. Nat. Acad. Sci. USA.

[109] Eckert H., LaVallee B., Schweiger B.J., Kinney A.J., Cahoon E.B., Clemente T. (2006). Co-expression of the borage Δ6 desaturase and the *Arabidopsis* Δ15 desaturase results in high accumulation of stearidonic acid in the seeds of transgenic soybean. Planta.

[110] Liu H.L., Yin Z.J., Xiao L., Xu Y.N., Qu L.Q. (2012). Identification and evaluation of ω-3 fatty acid desaturase genes for hyperfortifying α-linolenic acid in transgenic rice seed. J. Exp. Bot..

[111] D’Angeli S., Matteucci M., Fattorini L., Gismondi A., Ludovici M., Canini A., Altamura M.M. (2016). OeFAD8, OeLIP and OeOSM expression and activity in cold-acclimation of *Olea europaea,* a perennial dicot without winter-dormancy. Planta.

[112] Hernandez M.L., Sicardo M.D., Martinez-Rivas J.M. (2016). Differential contribution of endoplasmic reticulum and chloroplast ω-3 fatty acid desaturase genes to the linolenic acid content of olive (*Olea europaea*) fruit. Plant Cell Physiol..

[113] Hernandez M.L., Lima-Cabello E., Alché J.D., Martínez-Rivas J.M., Castro A.J. (2020). Lipid Composition and Associated Gene Expression Patterns during Pollen Germination and Pollen Tube Growth in Olive (*Olea europaea* L.). Plant Cell Physiol..

[114] Peng Z.Y., Ruan J., Tian H.Y., Shan L., Meng J.J., Guo F., Li X. (2020). The family of peanut fatty acid desaturase genes and a functional analysis of four ω-3 AhFAD3 members. Plant Mol. Biol. Rep..

[115] Dmitriev A.A., Kezimana P., Rozhmina T.A., Zhuchenko A.A., Povkhova L.V., Pushkova E.N., Melnikova N.V. (2020). Genetic diversity of SAD and FAD genes responsible for the fatty acid composition in flax cultivars and lines. BMC Plant Biol..

[116] Liu K., Zhao S., Wang S., Wang H., Zhang Z. (2020). Identification and analysis of the FAD gene family in walnuts (*Juglans regia* L.) based on transcriptome data. BMC Genom..

[117] Torres-Franklin M.L., Repellin A., Huynh V.B., d’Arcy-Lameta A., Zuily-Fodil Y., Pham-Thi A.T. (2009). Omega-3 fatty acid desaturase (FAD3, FAD7, FAD8) gene expression and linolenic acid content in cowpea leaves submitted to drought and after rehydration. Environ. Exp. Bot..

[118] Zhang Z., Jin X., Liu Z., Zhang J., Liu. W. (2020). Genome-wide identification of FAD gene family and functional analysis of MsFAD3.1 involved in the accumulation of α-linolenic acid in alfalfa. Crop Sci..

[119] Li J., Galla A.L., Avila C.A., Flattmann K., Vaughn K.L., Goggin F. (2021). Fatty acid desaturases in the chloroplast and endoplasmic reticulum promote susceptibility to the green peach aphid, myzus persicae, in *Arabidopsis thaliana*. Mol. Plant Microbe Interact.

[120] Adhikari N.D., Bates P.D., Browse J. (2016). Wrinkled1 rescues feedback inhibition of fatty acid synthesis in hydroxylase-expressing seeds. Plant Physiol..

[121] Bates P.D., Johnson S.R., Cao X., Li J., Nam J.W., Jaworski J.G., Ohlrogge J.B., Browse J. (2014). Fatty acid synthesis is inhibited by inefficient utilization of unusual fatty acids for glycerolipid assembly. Proc. Natl. Acad. Sci. USA.

[122] Bhattacharya S., Sinha S., Das N., Maiti M.K. (2015). Increasing the stearate content in seed oil of Brassica juncea by heterologous expression of MlFatB affects lipid content and germination frequency of transgenic seeds. Plant Physiol. Biochem..

[123] Cahoon E.B., Dietrich C.R., Meyer K., Damude H.G., Dyer J.M., Kinney A.J. (2006). Conjugated fatty acids accumulate to high levels in phospholipids of metabolically engineered soybean and *Arabidopsis* seeds. Phytochemistry.

[124] Cahoon E.B., Carlson T.J., Ripp K.G., Schweiger B.J., Cook G.A., Hall S.E., Kinney A.J. (1999). Biosynthetic origin of conjugated double bonds: Production of fatty acid components of high-value drying oils in transgenic soybean embryos. Proc. Natl. Acad. Sci. USA.

[125] Li R., Yu K., Wu Y., Tateno M., Hatanaka T., Hildebrand D.F. (2012). Vernonia DGATs can complement the disrupted oil and protein metabolism in epoxygenase-expressing soybean seeds. Metab. Eng..

[126] Eccleston V.S., Ohlrogge J.B. (1998). Expression of lauroyl-acyl carrier protein thioesterase in *Brassica napus* seeds induces pathways for both fatty acid oxidation and biosynthesis and implies a set point for triacylglycerol accumulation. Plant Cell.

[127] Yeom W.W., Kim H.J., Lee K., Cho H.S., Kim J., Jung H.W., Oh S., Jun S.E., Kim H.U., Chung Y. (2020). Increased Production of α-Linolenic Acid in Soybean Seeds by Overexpression of Lesquerella FAD3-1. Front. Plant Sci..

[128] Thompson J.D., Gibson T.J., Plewniak F., Jeanmougin F., Higgins D.G. (1997). The CLUSTAL_X windows interface: Flexible strategies for multiple sequence alignment aided by quality analysis tools. Nucl. Acids Res..

[129] Felsenstein J. (1985). Confidence limits on phylogenies: An approach using the bootstrap. Evolution.

[130] Zuckerkandl E., Pauling L., Bryson V., Vogel H.J. (1965). Evolutionary divergence and convergence in proteins. Evolving Genes and Proteins.

[131] Higgins D.G., Sharp P.M. (1988). CLUSTAL: A package for performing multiple sequence alignment on a microcomputer. Gene.

[132] Tamura K., Stecher G., Peterson D., Filipski A., Kumar S. (2013). MEGA6: Molecular evolutionary genetics analysis version 6.0. Mol. Biol. Evol..

[133] Hu B., Jin J., Guo A.Y., Zhang H., Luo J., Gao G. (2015). GSDS 2.0: An upgraded gene feature visualization server. Bioinformatics.

[134] Marchler-Bauer A., Bryant S.H. (2004). CD-search: Protein domain annotations on the fly. Nucl. Acids Res..

[135] Krogh A., von Heijne B.L., Sonnhammer E.L. (2001). Predicting transmembrane protein topology with a hidden Markov model: Application to complete genomes. J. Mol. Biol..

[136] Hirokawa T., Boon-Chieng S., Mitaku S. (1998). SOSUI: Classification and secondary structure prediction system for membrane proteins. Bioinformatics.

[137] Yu C.S., Chen Y.C., Lu C.H., Hwang J.K. (2006). Prediction of protein subcellular localization. Proteins Struct. Funct. Bioinf..

[138] Yoo S., Cho Y., Sheen J. (2007). *Arabidopsis* mesophyll protoplasts: A versatile cell system for transient gene expression analysis. Nat. Prot..

[139] Nelson B.K., Cai X., Nebenführ A. (2007). A multicolored set of in vivo organelle markers for colocalization studies in *Arabidopsis* and other plants. Plant J..

[140] Shao G., Wei X., Chen M., Tang S., Luo J., Jiao G., Hu P. (2012). Allelic variation for a candidate gene for GS7, responsible for grain shape in rice. Theor. Appl. Genet..

[141] Tang S.Q., Shao G.N., Wei X.J., Chen M.L., Sheng Z.H., Luo J., Hu P.S. (2013). QTL mapping of grain weight in rice and the validation of the QTL qTGW3.2. Gene.

[142] Yin C., Li H., Li S., Xu L., Zhao Z., Wang J. (2015). Genetic dissection on rice grain shape by the two-dimensional image analysis in one japonica × indica population consisting of recombinant inbred lines. Theor. Appl. Genet..

