# Supplementary material for: In Silico Analysis of Fatty Acid Desaturases Structures in Camelina sativa, and Functional Evaluation of Csafad7 and Csafad8 on Seed Oil Formation and Seed Morphology
Source: Int J Mol Sci. 2021 Oct 8;22(19):10857. doi: 10.3390/ijms221910857 (PMC8532002; doi:10.3390/ijms221910857)
Supplement: Supplementary file 1 [file ijms-22-10857-s001.zip › Figure S1.pdf]

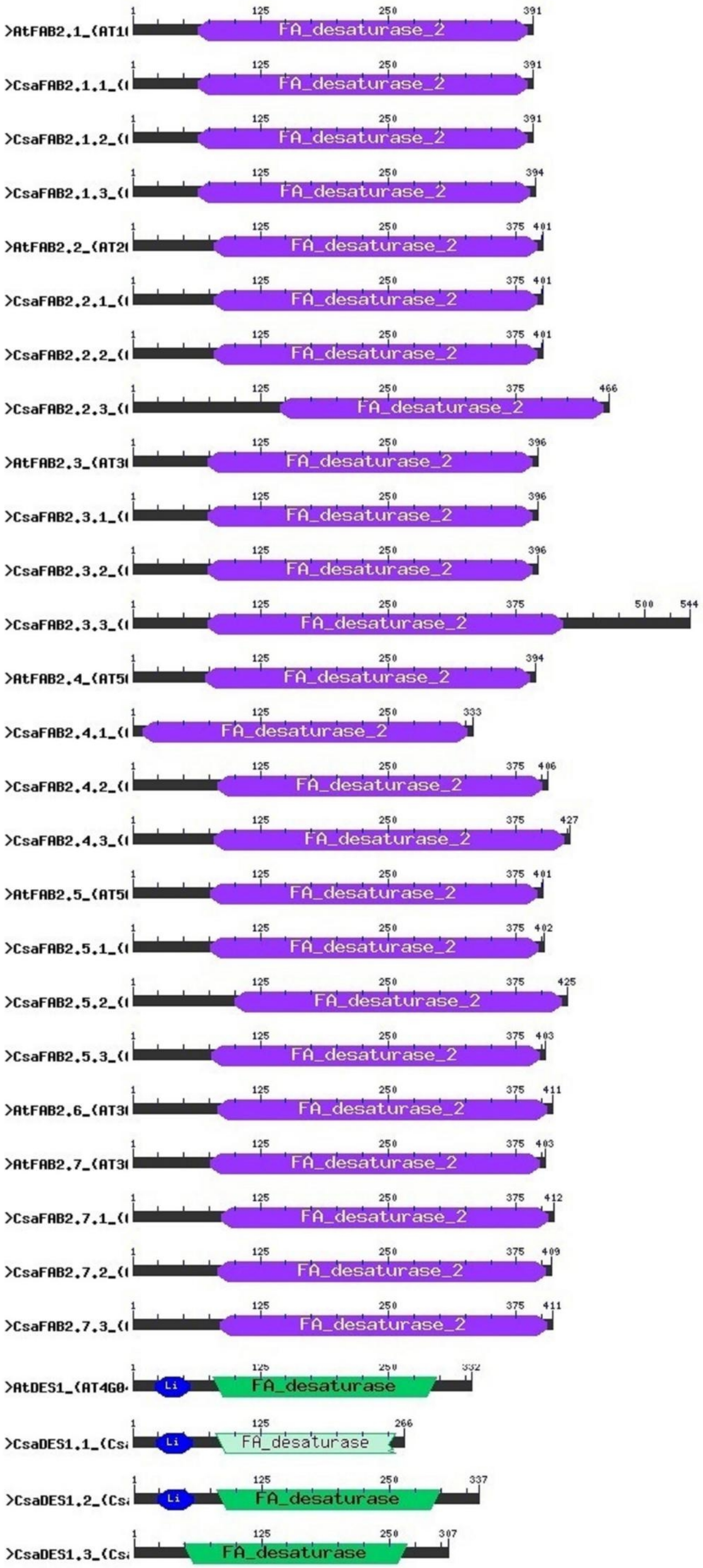
>AtFAB2.1_(AT1(
FA_desaturase_2
>CsaFAB2.1.1_((
FA_desaturase_2
>CsaFAB2.1.2_((
FA_desaturase_2
>CsaFAB2.1.3_((
FA_desaturase_2
>AtFAB2.2_(AT2(
FA_desaturase_2
>CsaFAB2.2.1_((
FA_desaturase_2
>CsaFAB2.2.2_((
FA_desaturase_2
>CsaFAB2.2.3_((
FA_desaturase_2
>AtFAB2.3_(AT3(
FA_desaturase_2
>CsaFAB2.3.1_((
FA_desaturase_2
>CsaFAB2.3.2_((
FA_desaturase_2
>CsaFAB2.3.3_((
FA_desaturase_2
>AtFAB2.4_(AT5(
FA_desaturase_2
>CsaFAB2.4.1_((
FA_desaturase_2
>CsaFAB2.4.2_((
FA_desaturase_2
>CsaFAB2.4.3_((
FA_desaturase_2
>AtFAB2.5_(AT5(
FA_desaturase_2
>CsaFAB2.5.1_((
FA_desaturase_2
>CsaFAB2.5.2_((
FA_desaturase_2
>CsaFAB2.5.3_((
FA_desaturase_2
>AtFAB2.6_(AT3(
FA_desaturase_2
>AtFAB2.7_(AT3(
FA_desaturase_2
>CsaFAB2.7.1_((
FA_desaturase_2
>CsaFAB2.7.2_((
FA_desaturase_2
>CsaFAB2.7.3_((
FA_desaturase_2
>AtDES1_(AT4G0
Li
FA_desaturase
>CsaDES1.1_(Cs
Li
FA_desaturase
>CsaDES1.2_(Cs
Li
FA_desaturase
>CsaDES1.3_(Cs
FA_desaturase

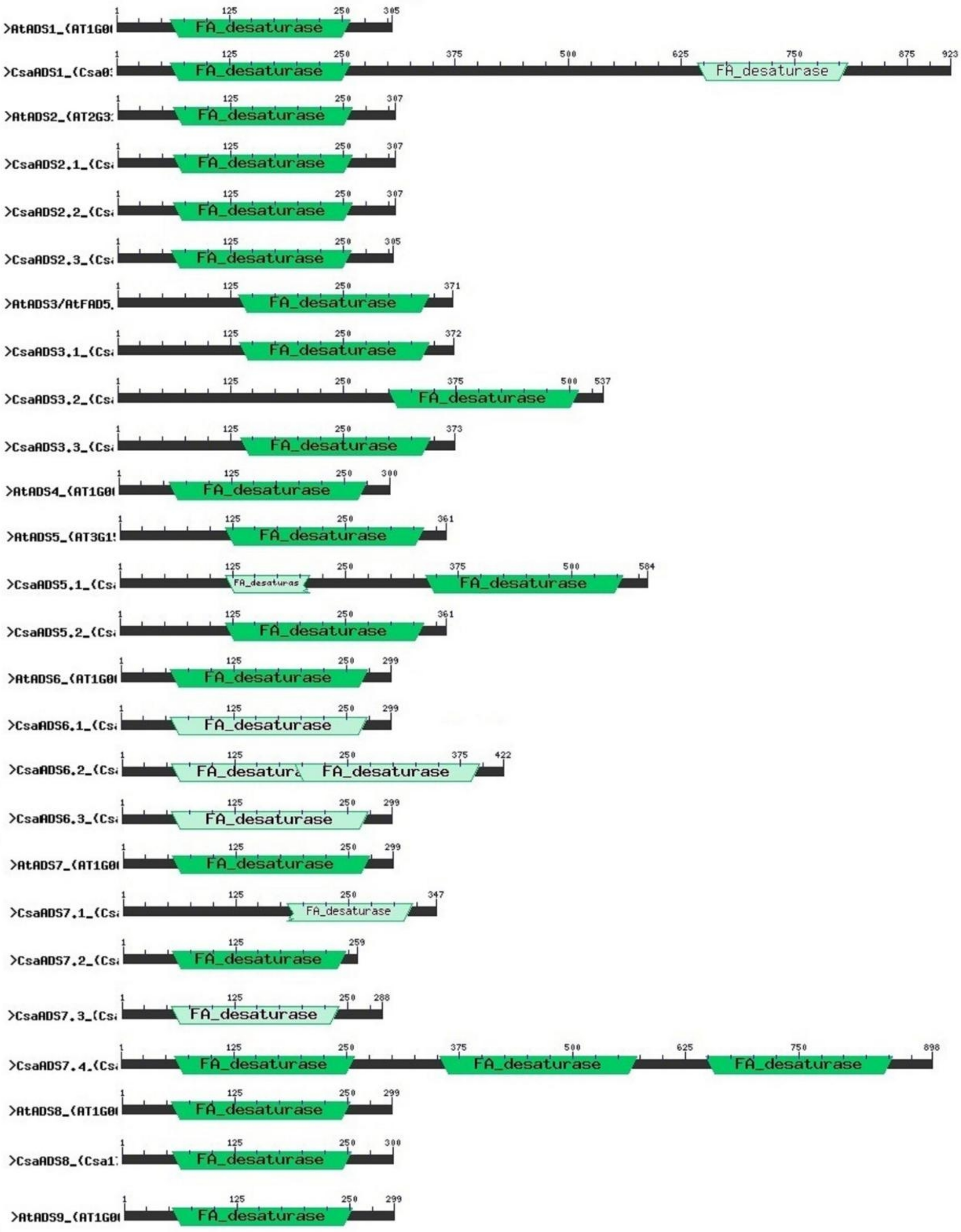

>AtADS1_(AT1G0
>CsaADS1_(Csa0
>AtADS2_(AT2G3
>CsaADS2.1_(Cs
>CsaADS2.2_(Cs
>CsaADS2.3_(Cs
>AtADS3/AtFAD5
>CsaADS3.1_(Cs
>CsaADS3.2_(Cs
>CsaADS3.3_(Cs
>AtADS4_(AT1G0
>AtADS5_(AT3G1
>CsaADS5.1_(Cs
>CsaADS5.2_(Cs
>AtADS6_(AT1G0
>CsaADS6.1_(Cs
>CsaADS6.2_(Cs
>CsaADS6.3_(Cs
>AtADS7_(AT1G0
>CsaADS7.1_(Cs
>CsaADS7.2_(Cs
>CsaADS7.3_(Cs
>CsaADS7.4_(Cs
>AtADS8_(AT1G0
>CsaADS8_(Csa1
>AtADS9_(AT1G0
FA_desaturase

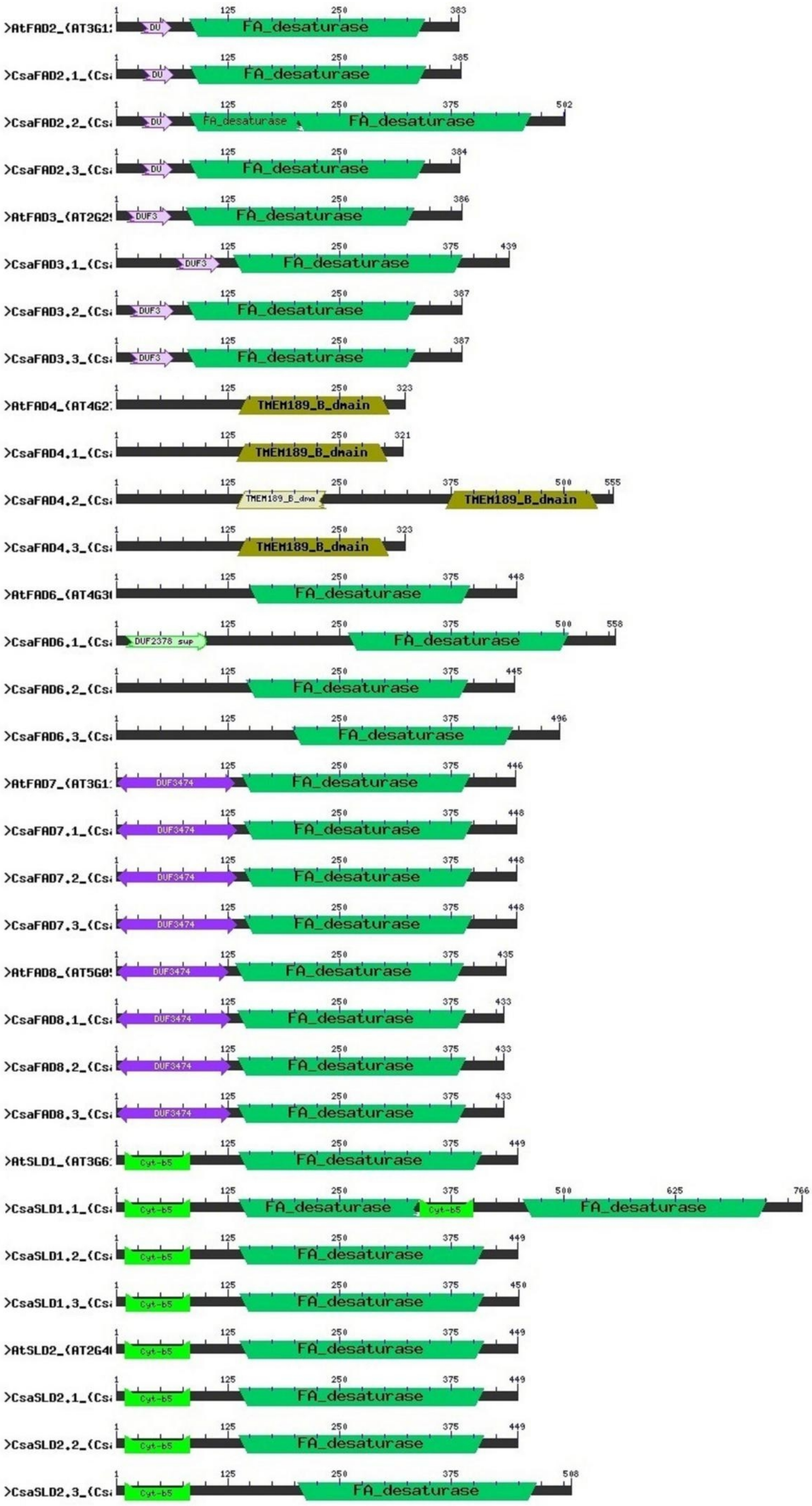
>AtFAD2_(AT3G1: DU FA_desaturase
>CsaFAD2.1_(Csa DU FA_desaturase
>CsaFAD2.2_(Csa DU FA_desaturase FA_desaturase
>CsaFAD2.3_(Csa DU FA_desaturase
>AtFAD3_(AT2G2 DUF3 FA_desaturase
>CsaFAD3.1_(Csa DUF3 FA_desaturase
>CsaFAD3.2_(Csa DUF3 FA_desaturase
>CsaFAD3.3_(Csa DUF3 FA_desaturase
>AtFAD4_(AT4G2 TMEM189_B_dmain
>CsaFAD4.1_(Csa TMEM189_B_dmain
>CsaFAD4.2_(Csa TMEM189_B_dma TMEM189_B_dmain
>CsaFAD4.3_(Csa TMEM189_B_dmain
>AtFAD6_(AT4G3 FA_desaturase
>CsaFAD6.1_(Csa DUF2378 sup FA_desaturase
>CsaFAD6.2_(Csa FA_desaturase
>CsaFAD6.3_(Csa FA_desaturase
>AtFAD7_(AT3G1 DUF3474 FA_desaturase
>CsaFAD7.1_(Csa DUF3474 FA_desaturase
>CsaFAD7.2_(Csa DUF3474 FA_desaturase
>CsaFAD7.3_(Csa DUF3474 FA_desaturase
>AtFAD8_(AT5G0 DUF3474 FA_desaturase
>CsaFAD8.1_(Csa DUF3474 FA_desaturase
>CsaFAD8.2_(Csa DUF3474 FA_desaturase
>CsaFAD8.3_(Csa DUF3474 FA_desaturase
>AtSLD1_(AT3G6 Cyt-b5 FA_desaturase
>CsaSLD1.1_(Csa Cyt-b5 FA_desaturase Cyt-b5 FA_desaturase
>CsaSLD1.2_(Csa Cyt-b5 FA_desaturase
>CsaSLD1.3_(Csa Cyt-b5 FA_desaturase
>AtSLD2_(AT2G4 Cyt-b5 FA_desaturase
>CsaSLD2.1_(Csa Cyt-b5 FA_desaturase
>CsaSLD2.2_(Csa Cyt-b5 FA_desaturase
>CsaSLD2.3_(Csa Cyt-b5 FA_desaturase

Figure S1 Domain architecture of fatty acid desaturases in *A. thaliana* and *C. sativa*. The architecture was generated by using Batch CD-search from NCBI database, using CDSEARCH/oasis_pfam v3 and E-value cut-off of 0.10.
