# Supplementary material for: In Silico Analysis of Fatty Acid Desaturases Structures in Camelina sativa, and Functional Evaluation of Csafad7 and Csafad8 on Seed Oil Formation and Seed Morphology
Source: Int J Mol Sci. 2021 Oct 8;22(19):10857. doi: 10.3390/ijms221910857 (PMC8532002; doi:10.3390/ijms221910857)
Supplement: Supplementary file 1 [file ijms-22-10857-s001.zip › Figure S2.pdf]

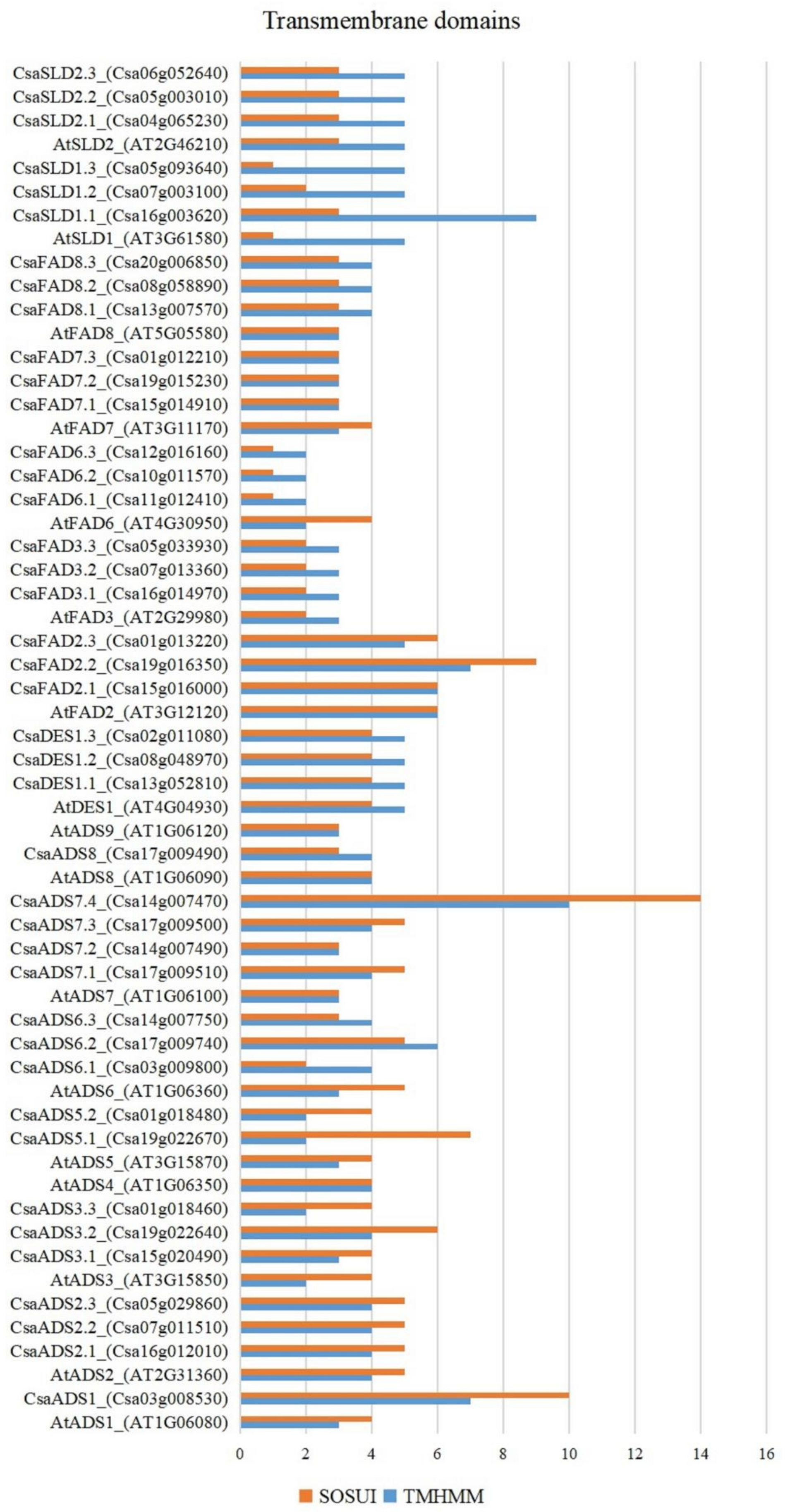

Figure S2 Number of transmembrane domains detected in fatty acid desaturases in *A. thaliana* and *C. sativa.*
