# Supplementary material for: In Silico Analysis of Fatty Acid Desaturases Structures in Camelina sativa, and Functional Evaluation of Csafad7 and Csafad8 on Seed Oil Formation and Seed Morphology
Source: Int J Mol Sci. 2021 Oct 8;22(19):10857. doi: 10.3390/ijms221910857 (PMC8532002; doi:10.3390/ijms221910857)
Supplement: Supplementary file 1 [file ijms-22-10857-s001.zip › Figure S3.pdf]

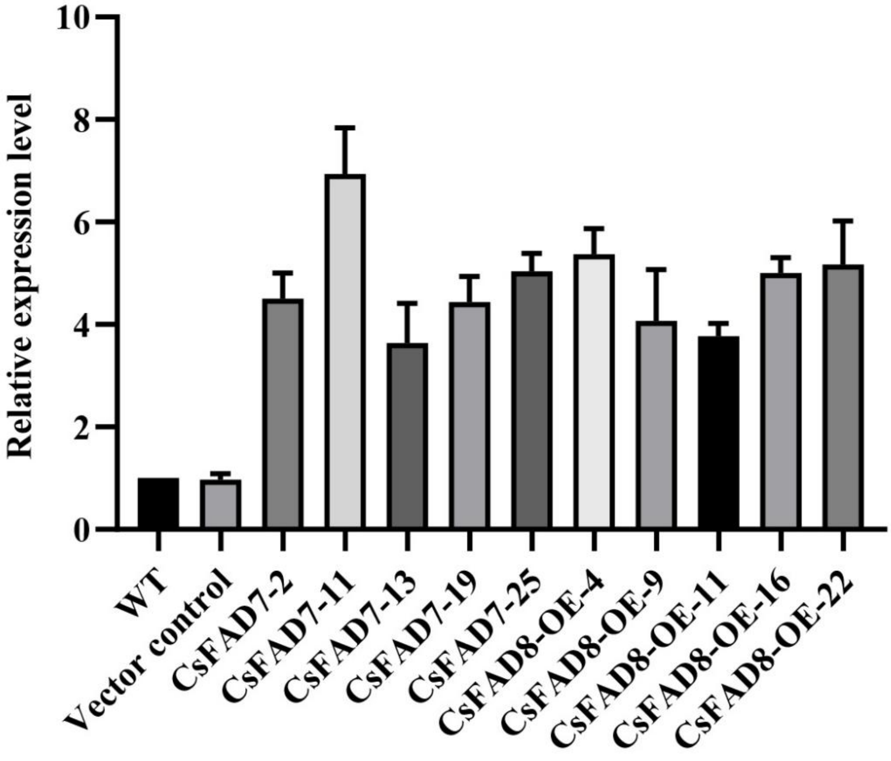


**Figure S3 Relative expression level of *CsaFAD7* and *CsaFAD8* in the transgenic lines, compared to the wild-type and the empty vector control group.** The developing siliques 5 days after flowering of transgenic lines and WT were collected for the qRT-PCR analysis and *Actin7* was applied as internal control. The results showed that the expression levels of *CsaFAD7* and *CsaFAD8* were significantly higher than those of WT and empty vector control group. While there was no significant difference between CsaFAD7-OE and CsaFAD8-OE.
