# Supplementary material for: In Silico Analysis of Fatty Acid Desaturases Structures in Camelina sativa, and Functional Evaluation of Csafad7 and Csafad8 on Seed Oil Formation and Seed Morphology
Source: Int J Mol Sci. 2021 Oct 8;22(19):10857. doi: 10.3390/ijms221910857 (PMC8532002; doi:10.3390/ijms221910857)
Supplement: Supplementary file 1 [file ijms-22-10857-s001.zip › Figure S4.pdf]

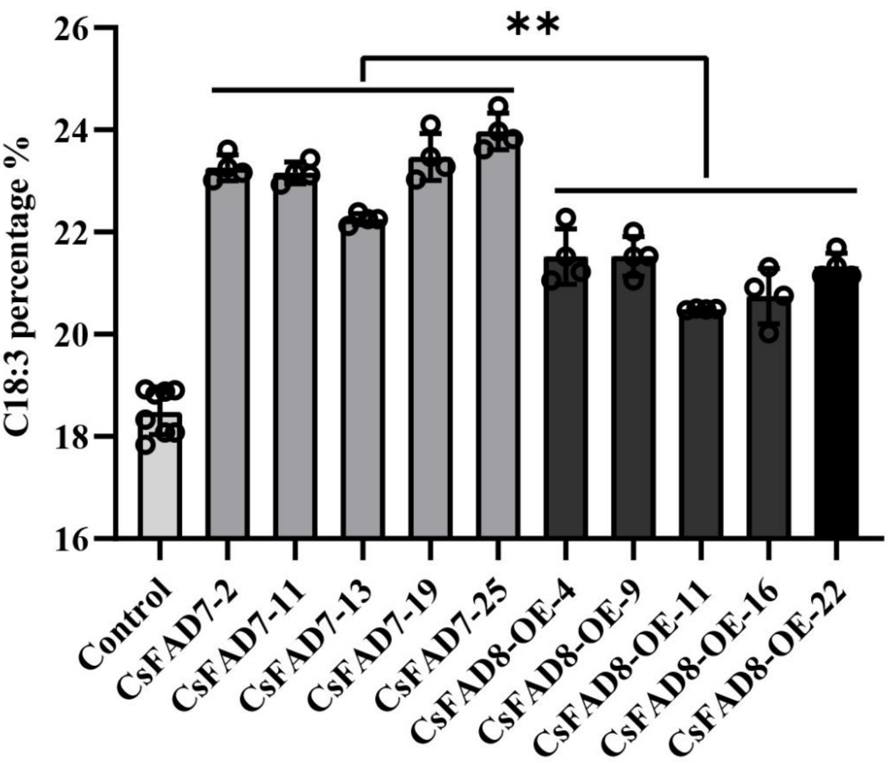


**Figure S4 Level of C18:3 in the *CsaFAD7* and *CsaFAD8* transgenic lines.** The *P* value is less than 0.01 between FAD7-OE and FAD8-OE transgenic lines which suggest the significant difference between the two data sets. Error bars indicate standard deviations. Value represents the mean value ± SD. Double asterisk represents significant difference (n=4, $p<0.01$).
