# Supplementary material for: In Silico Analysis of Fatty Acid Desaturases Structures in Camelina sativa, and Functional Evaluation of Csafad7 and Csafad8 on Seed Oil Formation and Seed Morphology
Source: Int J Mol Sci. 2021 Oct 8;22(19):10857. doi: 10.3390/ijms221910857 (PMC8532002; doi:10.3390/ijms221910857)
Supplement: Supplementary file 1 [file ijms-22-10857-s001.zip › Figure S5.pdf]

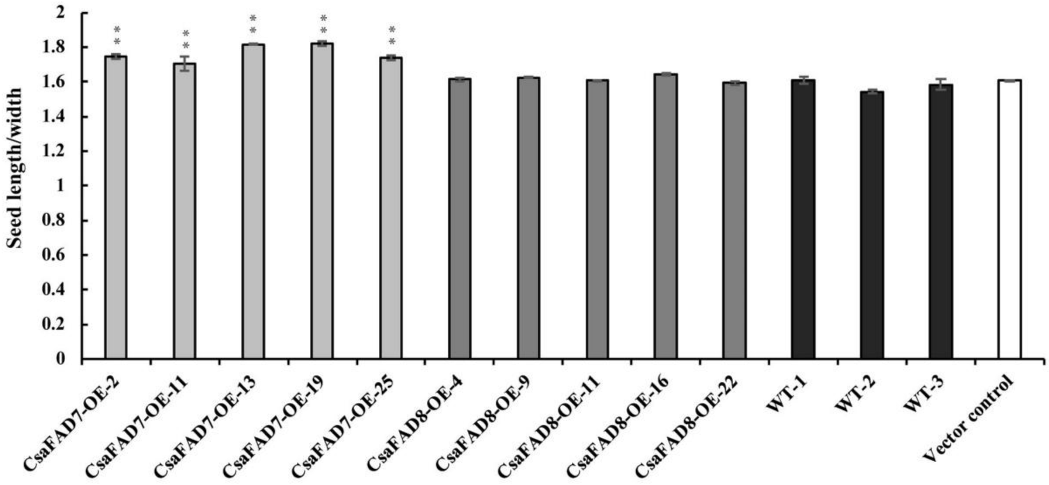

Figure S5 Seed length and width ratio of *CsaFAD7* and *CsaFAD8* overexpression lines of dry seeds. The dry mature seed size was measured with 5 times repeat for each line. Asterisk represents a significant difference ($P<0.01$, $n=5$). Vector control, *pBinGlyRed3* transgenic lines under wild type background as control.
